# Co-dynamics of COVID-19 and TB with COVID-19 vaccination and exogenous reinfection for TB: An optimal control application

**DOI:** 10.1016/j.idm.2023.05.005

**Published:** 2023-05-31

**Authors:** Zenebe Shiferaw Kifle, Legesse Lemecha Obsu

**Affiliations:** Department of Mathematics, Adama Science and Technology University, Adama, Ethiopia

**Keywords:** COVID-19, Co-infection, Exogenous reinfection, Basic reproduction number, Bifurcation, Optimal control

## Abstract

COVID-19 and Tuberculosis (TB) are among the major global public health problems and diseases with major socioeconomic impacts. The dynamics of these diseases are spread throughout the world with clinical similarities which makes them difficult to be mitigated. In this study, we formulate and analyze a mathematical model containing several epidemiological characteristics of the co-dynamics of COVID-19 and TB. Sufficient conditions are derived for the stability of both COVID-19 and TB sub-models equilibria. Under certain conditions, the TB sub-model could undergo the phenomenon of backward bifurcation whenever its associated reproduction number is less than one. The equilibria of the full TB-COVID-19 model are locally asymptotically stable, but not globally, due to the possible occurrence of backward bifurcation. The incorporation of exogenous reinfection into our model causes effects by allowing the occurrence of backward bifurcation for the basic reproduction number *R*_0_ < 1 and the exogenous reinfection rate greater than a threshold (*η* > *η*∗). The analytical results show that reducing *R*_0_ < 1 may not be sufficient to eliminate the disease from the community. The optimal control strategies were proposed to minimize the disease burden and related costs. The existence of optimal controls and their characterization are established using Pontryagin's Minimum Principle. Moreover, different numerical simulations of the control induced model are carried out to observe the effects of the control strategies. It reveals the usefulness of the optimization strategies in reducing COVID-19 infection and the co-infection of both diseases in the community.

## 1 Introduction

Coronavirus disease 2019 (COVID-19) and Tuberculosis (TB) are two major infectious diseases that pose a significant public health threat, and their co-infection exacerbates the situation. Both are contagious diseases that attack primarily the lungs. They share plenty of common symptoms such as fever, cough, and difficulty breathing. However, TB have been known with a longer incubation period with a slower onset of the disease (WHO, 2020b). COVID-19 is a communicable disease caused by a family of novel coronavirus called SARS-CoV-2 while TB is a respiratory pathogens caused by *Mycobacterium Tuberculosis* (MTB), which is transmitted between humans through the respiratory route such as cough, sneeze, speak, kiss or spit and most commonly affects the lungs, but can damage any tissue. TB can spread from active pulmonary TB persons but not latent TB. Worldwide, TB remains a leading cause of death. According to the World Health Organization Global TB Report 2021, approximately 10 million people contracted TB and 1.5 million died from TB in 2020, worldwide (WHO, 2021). COVID-19, on the other hand, about 480 million confirmed cases were reported up to the end of March 2022 and more than 6 million people were died due to COVID-19 worldwide (WHO, 2023). Currently, there is an emerging evidence that patients with latent TB and active TB disease are more likely to get SARS-CoV-2 and become severely infected with COVID-19 (Chen et al., 2020).

In the study of infectious diseases, co-epidemic requires critical understanding of how the diseases are related, prevalence, their prevention and treatment to effectively control these diseases. A co-epidemic occurs when the spread of one infectious disease stimulates that of another. This is just recently occurred with COVID-19 and TB. Different studies have been conducted on the co-interactions of COVID-19 and TB diseases (Chen et al., 2020; Davies, 2020; Motta et al., 2020; Mousquer, Peres, & Fiegenbaum, 2021; Petrone et al., 2021). In particular, Chen et al. (2020), observed that TB can increase the susceptibility to COVID-19 and the severity of its symptoms. In the study by Petrone et al. (2021), it is observed that peoples infected with COVID-19 and TB develop a weak immune response to SARS-COV-2. The work done in Mousquer et al. (2021) shows that COVID-19 and TB co-infected peoples are associated with higher morbidity and deaths. Besides, it is reported that patients having the past history of TB infection have high risk of mortality if they acquired COVID-19 (Davies, 2020). In another work done by Chen et al. (2020), patients having latent and active TB infection would have higher risk of COVID-19 infection with its severity.

Mathematical modeling has become the powerful tool for studying the behaviour of infectious diseases (Egonmwan & Okuonghae, 2019; Omame, Okuonghae, Umana, & Inyama, 2020; Omame, Okuonghae, Nwafor, & Odionyenma, 2021). Different mathematical modeling of COVID-19 with vaccination is studied in (Gumel, Iboi, Ngonghala, & Ngwa, 2020; L. Zhang, Ullah, Al Alwan, Alshehri, & Sumelka, 2021; Diagne, Rwezaura, Tchoumi, & Tchuenche, 2021; Yavuz, Coşar, Günay, & Özdemir, 2021). For instance, Gumel et al. (2020) present a mathematical analysis for the effects of vaccination and non-pharmaceutical interventions on COVID-19 dynamics. Their findings suggests that, adding the impacts of the therapeutic benefits of the vaccination against COVID-19 into the model resulted in a dramatic elimination of the pandemic. Diagne et al. (2021) developed and analyzed a mathematical model of COVID-19 with vaccination and treatment. Their results show the effectiveness of vaccination and treatment in reducing the spread of COVID-19, and more efforts are needed to eliminate the disease. They concluded that the combined or simultaneous use of non-pharmaceutical protective measures such as face masks, hand washing, and social distancing should continue to be encouraged. Furthermore, the effects of vaccination and other constant and time-dependent variable control measures on the dynamics of COVID-19 is investigated in (L. Zhang et al., 2021). Their findings revealed that, compared to constant controls, the time-depended control measures play a vital role in disease eradication. Moreover, mathematical models for the dynamics of COVID-19 and its co-infection with other diseases have been also developed (Hezam, Foul, & Alrasheedi, 2021; Omame, Sene, et al., 2021; Tchoumi, Diagne, Rwezaura, & Tchuenche, 2021).

Optimal controls have many applications in dynamical systems including systems governed by nonlinear ordinary differential equations. It can be seen as a control strategy in control theory (Ross, 2015). Mathematical models with optimal control analysis have become an important tool for understanding disease transmission dynamics and in the process of making decision regarding disease control. For example in the study of Malaria (Cai, Li, Tuncer, Martcheva, & Lashari, 2017; Makinde & Okosun, 2011; Mohammed-Awel & Gumel, 2019), TB (Das, Khajanchi, & Kar, 2020; Kim, Aurelio, & Jung, 2018), COVID-19 (Lemecha Obsu & Feyissa Balcha, 2020; Mondal & Khajanchi, 2022), HIV-TB coinfection (Aggarwal et al., 2020a; Silva & Torres, 2015), Malaria-COVID-19 coinfection (Tchoumi et al., 2021), COVID-19 and Diabetes coinfection (Omame, Sene, et al., 2021) and the references there in. Recently, some mathematical modeling of COVID-19 and TB co-infection with optimal control have been proposed and analyzed (Goudiaby et al., 2022; Mekonen, Obsu, & Habtemichael, 2022; Rwezaura, Diagne, Omame, de Espindola, & Tchuenche, 2022). In particular, Goudiaby et al. (2022) developed and studied a deterministic compartmental model for the transmission dynamics of tuberculosis and COVID-19 with optimal control. They suggest that the best strategy of interest to public health policy and decision makers to contain the spread of COVID-19 is to focus on prevention, treatment, and control of co-infection with COVID-19 which also results a lower percentage of total cost in COVID-19 prevention. In Mekonen et al. (2022), the authors have proposed an optimal control model of COVID-19 and TB by introducing four control measures (preventions and treatments for both diseases) to optimally manage the diseases. They showed that the prevalence of the co-infection reduced when concurrently all the control measures were implemented. On the other hand, Rwezaura et al. (2022) proposed the co-infection model for SARS-CoV-2 and TB that incorporates the vaccinated population with optimal control strategies to manage the spread of these two diseases. Their results suggest that either COVID-19 or TB control strategies will significantly mitigate a significant number of new co-infected cases.

However, the spread of COVID-19 and TB are still continuing and claiming more lives and thus, their co-dynamics need further investigation. Based on the descriptions above, the authors in this study are motivated in developing a mathematical model on the co-dynamics of COVID-19 and TB by incorporating COVID-19 vaccination and exogenous reinfection for TB. The importance of considering exogenous reinfection is justified in (Bandera et al., 2001; Caminero et al., 2001; Feng, Castillo-Chavez, & Capurro, 2000; van Rie et al., 1999). We also extend the model to an optimal control model by incorporating time variant controls to mitigate the spread of these two diseases. Compared to the previous COVID-19 and TB co-infection models, our model differ by several ways. For example, compared to the model proposed in (Goudiaby et al., 2022; Mekonen et al., 2022), our model considers the vaccination against COVID-19, TB re-infection after recovery, and exogenous TB re-infection. Moreover, compared to the model given in (Rwezaura et al., 2022), our model considers the exogenous TB re-infection.

The rest of this paper has been organized as follows. In Section 2, the details on the formulation of the proposed co-infection model are presented. The invariant region where the model is biological relevant is presented in Section 2.1. Sections 3.1, 3.2 present and theoretically examine the COVID-19 and TB sub-models, respectively. In Section 3.3, the full COVID-19 and TB co-infection model is analyzed. In Section 4, the extended optimal control model is presented and then analyzed using the well known Pontryagin's Maximum Principle. Moreover, different numerical simulations of control induced model are provided in Section 5. Finally, the paper concludes in Section 6.

## 2 Model formulation

In this section, the deterministic compartmental model of the transmission dynamics of both diseases are described. Depending on individuals’ disease status, at any time *t*, the total population *N*(*t*) is divided into nine compartments as described in the following Table 1.

**Table 1:** Model variables and their definitions.

Variables	Definitions
*S*	Unvaccinated susceptibles
*V*	Vaccinated susceptibles
*I*_*c*_	COVID-19 infected individuals
*R*_*c*_	Recovered from COVID-19
*L*	TB infected (in latent stage)
*I*_*t*_	TB infected (in active stage)
*R*_*t*_	TB recovered individuals
*I*_*cL*_	Individuals infected with latent TB and COVID-19
*I*_*tc*_	Individuals infected with active TB and COVID-19

The total population is given by

$$N\left(t\right)=S\left(t\right)+V\left(t\right)+I_{c}\left(t\right)+R_{c}\left(t\right)+L\left(t\right)+I_{t}\left(t\right)+R_{t}\left(t\right)+I_{cL}\left(t\right)+I_{tc}\left(t\right).$$

Individuals in the susceptible class are reduced at the rate,

$$\lambda_{c}=\left(1-\kappa \rho \right)\frac{\beta_{c}\left(I_{c}+\tau \left(I_{tc}+I_{cL}\right)\right)}{N}$$

when infected with COVID-19, and are also reduced at the rate

$$\lambda_{t}=\frac{\beta_{t}\left(I_{t}+I_{tc}\right)}{N}$$

when infected with TB. The forces of infection considered here are of standard incidence type (J. Zhang & Ma, 2003). The parameters *β*_*c*_ and *β*_*t*_ respectively represent the effective contact rate of COVID-19 and TB infected. The parameter *τ*(*τ* ≥ 1) accounts for the increased infectivity of co-infected individuals as a result of TB (Aggarwal & Raj, 2021; Rwezaura et al., 2022). The factor (1 − *κρ*) represents the effect of personal protection against COVID-19 (such as wearing a facial mask, physical distancing, hand washing with soap), where 0 < *ρ* < 1 is the efficacy of adopted protection strategy, and 0 < *κ* < 1 is compliance or the fraction of community employing it.

**Fig. 1:**
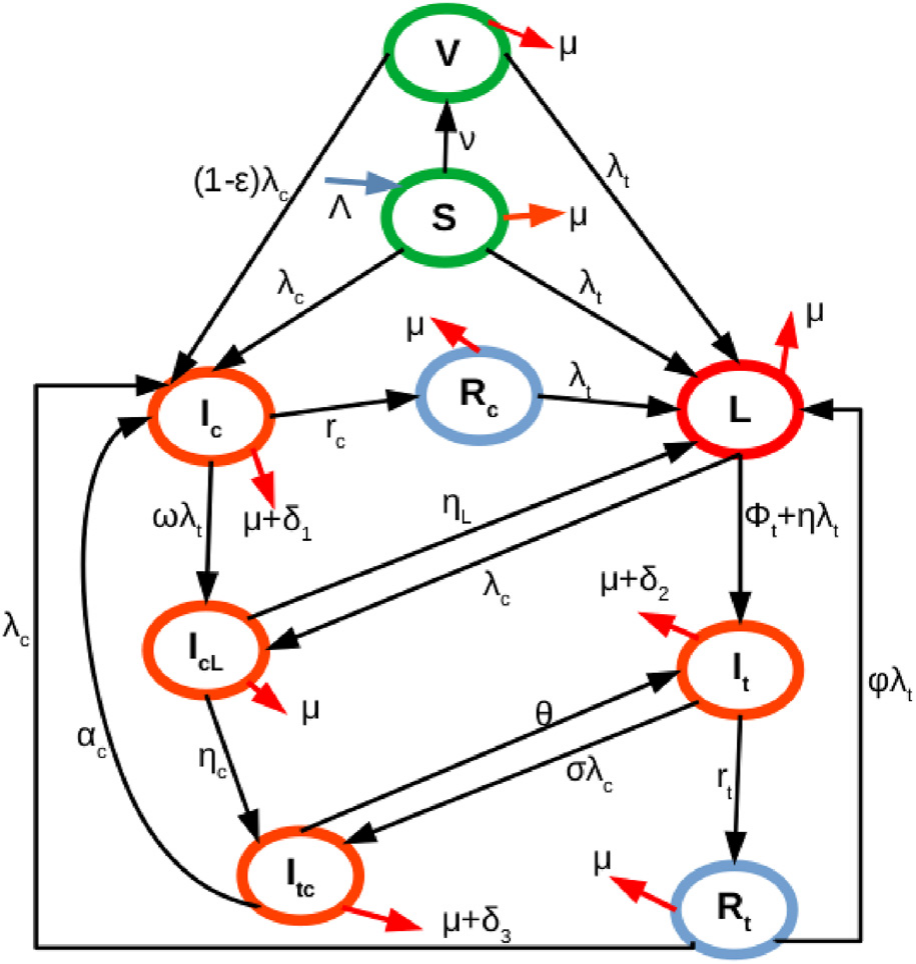
Flow diagram of the COVID-19 and TB co-infection model.

COVID-19 infected individuals may acquire TB infection at the rate *ωλ*_*t*_ and enter into the class *I*_*cL*_. The modification parameter *ω* (*ω* ≥ 1) is the enhancement factor accounting for the relative infectiousness of individuals acquiring TB following COVID-19 infection. Every individual after acquiring a TB infection, initially moves to the latently infected TB class, that is, in *L* or *I*_*cL*_, and then enters the active TB class (*I*_*t*_ or *I*_*tc*_) at a progression rate *φ*_*t*_ and *η*_*c*_, respectively. In addition, the latent TB infectives also move to the active TB infectious class, as exogenous reinfection occurs at a rate *ηλ*_*t*_ as a result of recent exposure of latent TB infectives with actively infected individuals. Individuals after recovery from TB again become susceptible to both diseases. They become COVID-19 infected at the rate *λ*_*c*_ and acquire TB infection at the rate *φλ*_*t*_. Such a transition is used in most TB and its co-dynamics models, where *φ*(0 ≤ *φ* ≤ 1) describes TB re-infection rate (Aggarwal et al., 2020a, 2020b; Aggarwal & Raj, 2021; Rwezaura et al., 2022). Individuals recovered from COVID-19 gains immunity against re-infection. However, there is possibility of infection with TB at the rate *λ*_*t*_. Latent TB infected individuals may acquire COVID-19 infection at the rate *λ*_*c*_ and enter into the class *I*_*cL*_. Also, active TB infected individuals may acquire COVID-19 infection at the rate *σλ*_*c*_ and enter into the class *I*_*tc*_. The modification parameter *σ*(*σ* ≥ 1) is represents the increasing factors for the relative infectiousness of individuals acquiring COVID-19 following TB infection. The parameters *η*_*L*_ and *θ* represents COVID-19 recovery rate for co-infected individuals in *I*_*cL*_ and *I*_*tc*_ classes, respectively. All other transitions in the model are described by the model flow diagram shown in Fig. 1. The definitions of the model parameters are provided in Table 2 together with their values.

**Table 2:** Model parameters and their definitions with values.

Parameters	Parameter description	Values	References
Λ	Recruitment rate	4696	Kifle & Obsu (2022)
*μ*	Natural death rate	1/(67.07 × 365)	Kifle & Obsu (2022)
*ν*	Vaccination rate	0.5482	Omame, Abbas, & Onyenegecha (2021)
*ε*	Vaccine efficacy	0.95	Rwezaura et al. (2022)
*κ*	Fraction of community employing COVID-19 prevention	0.3	Assumed
*ρ*	Efficacy of personal protection	0.5	Assumed
*δ*_1_	Death rate due to COVID-19	0.0029	Rwezaura et al. (2022)
*δ*_2_	Death rate due to TB	17/100000	TB-Treatment (2022)
*δ*_3_	COVID-19-TB related death rate	0.5140	Omame, Abbas, & Onyenegecha (2021)
*β*_*c*_	COVID-19 transmission rate	0.4531	Tchoumi et al. (2021)
*β*_*t*_	TB transmission rate	1.0	Assumed
*r*_*c*_	COVID-19 recovery rate for singly infected	0.023	Khan & Atangana (2022)
*r*_*t*_	TB recovery rate for singly infected	0.35	WHO (2020a)
*ω*	Enhancement factor of acquiring TB following COVID-19 infection	1.3	Rwezaura et al. (2022)
*σ*	Enhancement factor of acquiring COVID-19 following TB infection	1.3	Rwezaura et al. (2022)
*α*_*c*_	TB recovery rate for co-infected	0.012	Assumed
*φ*_*t*_	Rate of progression to active TB for latent infected	0.00002	Assumed
*η*_*c*_	Rate at which individuals leave *I*_*cL*_ class by becoming TB infectious	0.015	Rwezaura et al. (2022)
*η*_*L*_, *θ*	COVID-19 recovery rate for individuals in the *I*_*cL*_, *I*_*tc*_ classes respectively	0.02095	Omame, Abbas, & Onyenegecha (2021)
*τ*	Modification parameter	1.0	Rwezaura et al. (2022)
*φ*	TB re-infection rate	0.20	Omame, Abbas, & Onyenegecha (2021)
*η*	Exogenous re-infection rate	0.75	Assumed

From the aforementioned, the formulated model is

(1) $$\{\begin{array}{l} \begin{array}{c} \dot{S}=\Lambda -\left(\lambda_{c}+\lambda_{t}+\nu +\mu \right)S,\\ \dot{V}=\nu S-\left[\left(1-\varepsilon \right)\lambda_{c}+\mu +\lambda_{t}\right]V,\\ {\dot{I}}_{c}=\lambda_{c}\left[S+\left(1-\varepsilon \right)V+R_{t}\right]+\alpha_{c}I_{tc}-\left(r_{c}+\omega \lambda_{t}+\mu +\delta_{1}\right)I_{c},\\ \dot{R_{c}}=r_{c}I_{c}-\left(\lambda_{t}+\mu \right)R_{c},\\ \dot{L}=\lambda_{t}\left[S+V+R_{c}+\varphi R_{t}\right]+\eta_{L}I_{cL}-\eta \lambda_{t}L-\left(\varphi_{t}+\lambda_{c}+\mu \right)L,\\ {\dot{I}}_{t}=\varphi_{t}L+\eta \lambda_{t}L+\theta I_{tc}-\left(\sigma \lambda_{c}+r_{t}+\mu +\delta_{2}\right)I_{t},\\ \dot{R_{t}}=r_{t}I_{t}-\left(\lambda_{c}+\varphi \lambda_{t}+\mu \right)R_{t},\\ {\dot{I}}_{cL}=\omega \lambda_{t}I_{c}+\lambda_{c}L-\left(\eta_{c}+\eta_{L}+\mu \right)I_{cL},\\ {\dot{I}}_{tc}=\sigma \lambda_{c}I_{t}+\eta_{c}I_{cL}-\left(\alpha_{c}+\theta +\mu +\delta_{3}\right)I_{tc}, \end{array}\; \end{array}$$

with initial conditions

(2) $$\left\{\begin{array}{l} \begin{array}{cc} & S\left(0\right)\geq 0,\;V\left(0\right)\geq 0,\;I_{c}\left(0\right)\geq 0,\;R_{c}\left(0\right)\geq 0,\\ & L\left(0\right)\geq 0,\;I_{t}\left(0\right)\geq 0,\;R_{t}\left(0\right)\geq 0,\;I_{cL}\left(0\right)\geq 0,\;I_{tc}\left(0\right)\geq 0. \end{array}\; \end{array}\right)$$

The parameter *η* in the rate of exogenous reinfection term *ηλ*_*t*_*L* represents the reinfection level. When *η* = 0, model (1) reduces to the coinfection model with out exogenous reinfection. A value of *η* ∈ (0, 1) implies that reinfection is less likely than a new infection.

### 2.1 Basic properties of the model

#### 2.1.1 Positivity and boundedness of solutions

Since each component of the given model (1) represents the human population, we need to show all variables *S*(*t*), *V*(*t*), *I*_*c*_(*t*), *R*_*c*_(*t*), *L*(*t*), *I*_*t*_(*t*), *R*_*t*_(*t*), *I*_*cL*_(*t*) and *I*_*tc*_(*t*) are positive for all time *t* ≥ 0. Therefore, we state the following theorem:

Theorem 1 *All solutions of model* (1) *with nonnegative initial conditions remain nonnegative for all* *t* > 0*.*

*Proof*. For proving the positivity of all the components of the model (1), we assume on the contrary that, there exists a first time *t*_1_, such that

$$\min\left\{X\left(t_{1}\right)\right\}=0\;\text{and}\;\min\left\{X\left(t\right)\right\}>0,\text{for all}\;t\in \left[0,t_{1}\right).$$

Here, *X*(*t*) = *S*(*t*), *V*(*t*), *I*_*c*_(*t*), *R*_*c*_(*t*), *L*(*t*), *I*_*t*_(*t*), *R*_*t*_(*t*), *I*_*cL*_(*t*), *I*_*tc*_(*t*). Following our assumption, we first let,

$$\min\left\{X\left(t_{1}\right)\right\}=S\left(t_{1}\right).$$

Hence, *S*(*t*_1_) = 0 and *S*(*t*) > 0 for all $t\in \left(\left[0,t_{1}\right)\right)$. But,

$$\begin{array}{ccc} \frac{dS\left(t_{1}\right)}{dt} & =\Lambda -\left(\lambda_{c}+\lambda_{t}+\nu +\mu \right)S\left(t_{1}\right) & \\ & \geq \left(\lambda_{c}+\lambda_{t}+\nu +\mu \right)S\left(t_{1}\right), & \end{array}$$

from which we get,

$$S\left(t_{1}\right)\geq S\left(0\right)e^{\int_{0}^{t_{1}}\left(\lambda_{c}+\lambda_{t}+\nu +\mu \right)dr}>0,$$

which contradicts our assumption *S*(*t*_1_) = 0. Hence, *S*(*t*) > 0 for all *t* ≥ 0. Similarly, we can prove that all solution components are positive for *t* ≥ 0 in all other cases. □

#### 2.1.2 Invariant region

Theorem 2 *The closed region*

$$\Omega =\left\{\left(S,V,I_{c},R_{c},L,I_{t},R_{t},I_{cL},I_{tc}\right)\in R_{+}^{9}:N\left(t\right)\leq \frac{\Lambda }{\mu }\right\}.$$

*is a positively invariant set for the system* (1) *and attracts all positive solutions.*

*Proof*. The rate of change of total population for the model (1) is

$$\frac{dN}{dt}=\Lambda -\mu N-\left(\delta_{1}I_{c}+\delta_{2}I_{t}+\delta_{3}I_{tc}\right)\leq \Lambda -\mu N.$$

Thus, it follows that

$$N\left(t\right)\leq N\left(0\right)e^{-\mu t}+\frac{\Lambda }{\mu }\left(1-e^{-\mu t}\right).$$

More specifically, $0<N\left(t\right)\leq \frac{\Lambda }{\mu }$, if $N\left(0\right)\leq \frac{\Lambda }{\mu }$. That is, *N*(*t*) is bounded and all solutions beginning in Ω approach, enter, or remain in Ω. As a result, the model in (1) can be regarded as being well-posed. □

## 3 Model analysis

In this section, two sub-models, namely COVID-19 sub-model and TB sub-model are first considered.

### 3.1 COVID-19 sub-model

By setting *I*_*t*_(*t*) = *I*_*cL*_(*t*) = *I*_*tc*_(*t*) = *R*_*t*_(*t*) = 0 in model (1), we obtain the following COVID-19 only model

(3) $$\{\begin{array}{l} \begin{array}{cc} & \dot{S}=\Lambda -\left(\lambda_{c}+\nu +\mu \right)S,\\ & \dot{V}=\nu S-\left[\left(1-\varepsilon \right)\lambda_{c}+\mu \right]V,\\ & {\dot{I}}_{c}=\lambda_{c}\left[S+\left(1-\varepsilon \right)V\right]-\left(r_{c}+\mu +\delta_{1}\right)I_{c},\\ & \dot{R_{c}}=r_{c}I_{c}-\mu R_{c}, \end{array}\; \end{array}$$

where $\lambda_{c}=\left(1-\kappa \rho \right)\frac{\beta_{c}I_{c}}{N}$ is the force of infection and *N* = *S*(*t*) + *V*(*t*) + *I*_*c*_(*t*) + *R*_*c*_(*t*).

The COVID-19 sub-model (3) will be studied in the following feasible region

$$\Omega_{C}=\left\{\left(S,V,I_{c},R_{c}\right)\in R_{+}^{4}:N\left(t\right)\leq \frac{\Lambda }{\mu }\right\}.$$

Analogous to Theorem 2, Ω_*C*_ can be easily proved to be positively invariant.

#### 3.1.1 Basic reproduction numbers

The COVID-19 sub-model (3) has a disease-free equilibrium (DFE) given by

$$E_{c}^{0}=\left(\frac{\Lambda }{\nu +\mu },\frac{\Lambda \nu }{\mu \left(\nu +\mu \right)},0,0\right).$$

We can calculate the basic reproduction number using the next generation matrix operator (Van den Driessche & Watmough, 2002). Thus, following the same approach as in Appendix A, the *vaccination-induced* reproduction number of the COVID-19 sub-model (3) is given by

(4) $$R_{vC}=\frac{\left(1-\kappa \rho \right)\beta_{c}\left[\mu +\left(1-\varepsilon \right)\nu \right]}{\left(\nu +\mu \right)\left(r_{c}+\mu +\delta_{1}\right)}.$$

In the absence of COVID-19 vaccination (*ν* = 0), the basic reproduction number is given by

(5) $$R_{0C}=\frac{\left(1-\kappa \rho \right)\beta_{c}}{r_{c}+\mu +\delta_{1}}.$$

Note that

$$R_{vC}=R_{0C}\psi ,$$

where $\psi =\frac{\left[\mu +\left(1-\varepsilon \right)\nu \right]}{\nu +\mu }$. Since *ψ* < 1, as expected *R*_*vC*_ < *R*_0*C*_. The value of *ψ* becomes small with high value of vaccine efficacy ($\varepsilon \in \left(\left(0,1\right]\right)$). The parameter *ψ* which depends on the rate of vaccination and vaccine efficacy represents the effect of COVID-19 vaccine implementation in reducing the vaccine-induced reproduction number.

#### 3.1.2 Stability analysis of DFE

The local stability of DFE $\left(E_{c}^{0}\right)$ of COVID-19 sub-model (3) is determined using the eigenvalues of the Jacobian matrix at $E_{c}^{0}$, which is given by

$$J_{E_{c}^{0}}=\left(\begin{array}{cccc} -\left(\nu +\mu \right) & 0 & \frac{-\left(1-\kappa \rho \right)\beta_{c}\mu }{\nu +\mu } & 0\\ \nu & -\mu & \frac{-\left(1-\kappa \rho \right)\beta_{c}\left(1-\varepsilon \right)\nu }{\nu +\mu } & 0\\ 0 & 0 & \frac{\left(1-\kappa \rho \right)\beta_{c}\left(\mu +\left(1-\varepsilon \right)\nu \right)}{\nu +\mu }-\left(r_{c}+\mu +\delta_{1}\right) & 0\\ 0 & 0 & r_{c} & -\mu \end{array}\right).$$

The corresponding eigenvalues of the Jacobian matrix $J_{E_{c}^{0}}$ are

$$\lambda_{1}=-\left(\nu +\mu \right),\;\lambda_{2}=-\mu ,\;\lambda_{3}=\frac{\left(1-\kappa \rho \right)\beta_{c}\left(\mu +\left(1-\varepsilon \right)\nu \right)}{\nu +\mu }-\left(r_{c}+\mu +\delta_{1}\right),\;\text{and}\;\lambda_{4}=-\mu .$$

We can rewrite the eigenvalue *λ*_3_ as

$$\lambda_{3}=\frac{\left(1-\kappa \rho \right)\beta_{c}\left(\mu +\left(1-\varepsilon \right)\nu \right)}{\nu +\mu }-\left(r_{c}+\mu +\delta_{1}\right)=\left(r_{c}+\mu +\delta_{1}\right)\left(R_{vC}-1\right).$$

Since the eigenvalues *λ*_1_, *λ*_2_ and *λ*_4_ have negative real parts, the local stability of the DFE, $\left(E_{c}^{0}\right)$ depends on the sign of the eigenvalue *λ*_3_. Thus, the following result have been established.

Lemma 3 ***(Local stability of DFE:)****The DFE*$E_{c}^{0}$*of the COVID-19 sub-model* (3) *is locally asymptotically stable if* *R*_*vC*_ < 1 *and unstable if* *R*_*vC*_ > 1*.*

#### 3.1.3 Existence of endemic equilibrium for COVID-19 sub-model

For finding the endemic equilibrium point (EE) point for COVID-19 sub-model (3), we must solve the following system of equations at an arbitrary equilibrium point $E_{c}^{*}=\left(S^{*},V^{*},I_{c}^{*},R_{c}^{*}\right)$:

(6) $$\begin{array}{c} \Lambda -\left(\lambda_{c}^{*}+\nu +\mu \right)S^{*}=0\\ \nu S^{*}-\left[\left(1-\varepsilon \right)\lambda_{c}^{*}+\mu \right]V^{*}=0\\ \lambda_{c}^{*}\left[S^{*}+\left(1-\varepsilon \right)V^{*}\right]-k_{0}I_{c}^{*}=0\\ r_{c}I_{c}^{*}-\mu R_{c}^{*}=0, \end{array}$$

where $\lambda_{c}^{*}=\frac{\left(1-\kappa \rho \right)\beta_{c}I_{c}^{*}}{N^{*}}=\frac{\left(1-\kappa \rho \right)\beta_{c}I_{c}^{*}}{S^{*}+V^{*}+I_{c}^{*}+R_{c}^{*}}$ and *k*_0_ = *r*_*c*_ + *μ* + *δ*_1_.

After some algebraic calculation, the EE of COVID-19 sub-model (3) is given by

(7) $$\begin{array}{c} S^{*}=\frac{\Lambda }{\lambda_{c}^{*}+\nu +\mu },\\ V^{*}=\frac{\Lambda \nu }{\left(\lambda_{c}^{*}+\nu +\mu \right)\left(\left(1-\varepsilon \right)\lambda_{c}^{*}+\mu \right)},\\ I_{c}^{*}=\frac{\Lambda \lambda_{c}^{*}\left[\left(1-\varepsilon \right)\lambda_{c}^{*}+\mu +\left(1-\varepsilon \right)\nu \right]}{k_{0}\left(\lambda_{c}^{*}+\nu +\mu \right)\left(\left(1-\varepsilon \right)\lambda_{c}^{*}+\mu \right)},\\ R_{c}^{*}=\frac{\Lambda r_{c}\lambda_{c}^{*}\left[\left(1-\varepsilon \right)\lambda_{c}^{*}+\mu +\left(1-\varepsilon \right)\nu \right]}{\mu k_{0}\left(\lambda_{c}^{*}+\nu +\mu \right)\left(\left(1-\varepsilon \right)\lambda_{c}^{*}+\mu \right)}, \end{array}$$

where

(8) $$\lambda_{c}^{*}=\frac{\left(1-\kappa \rho \right)\beta_{c}I_{c}^{*}}{S^{*}+V^{*}+I_{c}^{*}+R_{c}^{*}}.$$

Note that

(9) $$\left(1-\kappa \rho \right)\beta_{c}I_{c}^{*}=\frac{\left(1-\kappa \rho \right)\beta_{c}\Lambda \lambda_{c}^{*}\left[\left(1-\varepsilon \right)\lambda_{c}^{*}+\mu +\left(1-\varepsilon \right)\nu \right]}{k_{0}\left[\lambda_{c}^{*}+\nu +\mu \right]\left[\left(1-\varepsilon \right)\lambda_{c}^{*}+\mu \right]}$$

and

(10) $$S^{*}+V^{*}+I_{c}^{*}+R_{c}^{*}=\Lambda \frac{\mu k_{0}\left[\left(1-\varepsilon \right)\lambda_{c}^{*}+\mu +\nu \right]+\lambda_{c}^{*}\left[\left(1-\varepsilon \right)\lambda_{c}^{*}+\mu +\left(1-\varepsilon \right)\nu \right]\left[\mu +r_{c}\right]}{\mu k_{0}\left[\lambda_{c}^{*}+\nu +\mu \right]\left[\left(1-\varepsilon \right)\lambda_{c}^{*}+\mu \right]}.$$

Substituting (9) and (10) into (8), we have

$$\lambda_{c}^{*}=\frac{\left(1-\kappa \rho \right)\beta_{c}\mu \left[\left(1-\varepsilon \right)\lambda_{c}^{*}+\mu +\left(1-\varepsilon \right)\nu \right]\lambda_{c}^{*}}{\mu k_{0}\left[\left(1-\varepsilon \right)\lambda_{c}^{*}+\mu +\nu \right]+\lambda_{c}^{*}\left[\left(1-\varepsilon \right)\lambda_{c}^{*}+\mu +\left(1-\varepsilon \right)\nu \right]\left[\mu +r_{c}\right]}.$$

This gives us

$$\lambda_{c}^{*}=0\;\text{or}\;\mu k_{0}\left[\left(1-\varepsilon \right)\lambda_{c}^{*}+\mu +\nu \right]+\lambda_{c}^{*}\left[\left(1-\varepsilon \right)\lambda_{c}^{*}+\mu +\left(1-\varepsilon \right)\nu \right]\left[\mu +r_{c}\right]-\left(1-\kappa \rho \right)\beta_{c}\mu \left[\left(1-\varepsilon \right)\lambda_{c}^{*}+\mu +\left(1-\varepsilon \right)\nu \right]=0.$$

Here, $\lambda_{c}^{*}=0$ gives the DFE, $E_{c}^{0}$. Rearranging the second equation gives us the following quadratic equation

(11) $$A{\left(\lambda_{c}^{*}\right)}^{2}+B\lambda_{c}^{*}+C=0,$$

where

(12) $$\begin{array}{c} A=\left(1-\varepsilon \right)\left(\mu +r_{c}\right)\\ B=\left(\mu +r_{c}\right)\left[\mu +\left(1-\varepsilon \right)\nu \right]+\mu k_{0}\left(1-\varepsilon \right)\left[1-R_{0C}\right]\\ C=\mu k_{0}\left(\nu +\mu \right)\left(1-R_{vC}\right). \end{array}$$

It is important to note that the coefficient *A* of the quadratic (11) is always positive. If *R*_0*C*_ < 1 (i.e., *R*_*vC*_ is also less than unity), then *B* > 0 and *C* > 0. In this case, since all coefficients of the quadratic equation (11) are positive, from Routh-Hurwitz criterion there is no positive root(s) of (11). If *R*_*vC*_ > 1, then *C* < 0 and *B* > 0 or *B* < 0. In this case, we have a unique positive root for the quadratic (11). Hence, the following result can be established:

Theorem 4 *The COVID-19 sub-model* (3) *has only one unique endemic equilibrium if* *R*_*vC*_ > 1*.*

#### 3.1.4 Bifurcation analysis

Depending on the threshold parameter *R*_*vC*_, the DFE can interchange its stability with the EE. We want to determine the direction of bifurcation (either forward or backward) using the centre manifold theory as described in Theorem 4.1 from (Castillo-Chavez & Song, 2004). The following variable changes and simplifications are first done in order to apply this theory.

Let $x={\left(x_{1},x_{2},x_{3},x_{4}\right)}^{T}={\left(S,V,I_{c},R_{c}\right)}^{T}$. Then we can rewrite the model (3) in the form $\frac{dx}{dt}=f\left(x\right)$ with $f\left(x\right)=\left(f_{1}\left(x\right),f_{2}\left(x\right),f_{3}\left(x\right),f_{4}\left(x\right)\right)$. That is,

(13) $$\left\{\begin{array}{l} \begin{array}{cc} & \frac{dx_{1}}{dt}=f_{1}\left(x\right)=\Lambda -\left(\lambda_{c}+\nu +\mu \right)x_{1},\\ & \frac{dx_{2}}{dt}=f_{2}\left(x\right)=\nu x_{1}-\left(\left(1-\varepsilon \right)\lambda_{c}+\mu \right)x_{2},\\ & \frac{dx_{3}}{dt}=f_{3}\left(x\right)=\lambda_{c}\left(x_{1}+\left(1-\varepsilon \right)x_{2}\right)-k_{0}x_{3},\\ & \frac{dx_{4}}{dt}=f_{3}\left(x\right)=r_{c}x_{3}-\mu x_{4}. \end{array}\; \end{array}\right)$$

where $\lambda_{c}=\frac{\left(1-\kappa \rho \right)\beta_{c}x_{3}}{x_{1}+x_{2}+x_{3}+x_{4}}$ and *k*_0_ = *r*_*c*_ + *μ* + *δ*_1_. Taking $\beta_{c}^{*}$ as the bifurcation parameter and setting *R*_*vC*_ = 1, we have

$$\beta_{c}=\beta_{c}^{*}=\frac{k_{0}\left(\nu +\mu \right)}{\left(1-\kappa \rho \right)\left(\mu +\left(1-\varepsilon \right)\nu \right)}.$$

The Jacobian matrix of the system (13) evaluated at $E_{c}^{0}$ and $\beta_{c}=\beta_{c}^{*}$ is given by

$$J_{\left(E_{c}^{0},\beta_{c}^{*}\right)}=\left(\begin{array}{cccc} -\left(\nu +\mu \right) & 0 & -\frac{\mu k_{0}}{\mu +\left(1-\varepsilon \right)\nu } & 0\\ \nu & -\mu & -\frac{\left(1-\varepsilon \right)\nu k_{0}}{\mu +\left(1-\varepsilon \right)\nu } & 0\\ 0 & 0 & 0 & 0\\ 0 & 0 & r_{c} & -\mu \end{array}\right).$$

The Jacobian $J_{\left(E_{c}^{0},\beta_{c}^{*}\right)}$ has an obvious simple zero eigenvalue, and all other eigenvalues have a negative sign. Hence, $E_{c}^{0}$ is a non-hyperbolic equilibrium, when $\beta_{c}=\beta_{c}^{*}$. Thus, the Castillo-Chavez and Song can be used to analyze the dynamics of the model (13) near $\beta_{c}=\beta_{c}^{*}$. By using the notation in Theorem 4.1 given in (Castillo-Chavez & Song, 2004), we proceed as follows.

*Right eigenvector:* Let $w={\left(w_{1},w_{2},w_{3},w_{4}\right)}^{T}$ be the right eigenvector associated to the zero eigenvalue. i.e.,

$$J_{\left(E_{c}^{0},\beta_{c}^{*}\right)}\cdot w=0.$$

Solving for *w*_1_, *w*_2_, *w*_3_ and *w*_4_, we get the right eigenvector $w={\left(w_{1},w_{2},w_{3},w_{4}\right)}^{T}$ such that

$$w_{1}=-\frac{\mu k_{0}}{\left(\nu +\mu \right)\left(\mu +\left(1-\varepsilon \right)\nu \right)}w_{3},\;w_{2}=-\frac{\nu k_{0}\left[\nu +\left(1-\varepsilon \right)\left(\nu +\mu \right)\right]}{\mu \left(\nu +\mu \right)\left(\mu +\left(1-\varepsilon \right)\nu \right)}w_{3},\;w_{4}=\frac{r_{c}}{\mu }w_{3},\;w_{3}=w_{3}>0.$$

*Left eigenvector:* Let *v* = (*v*_1_, *v*_2_, *v*_3_, *v*_4_) be the left eigenvector associated to the zero eigenvalue. i.e.,

$$v\cdot J_{\left(E_{c}^{0},\beta_{c}^{*}\right)}=0.$$

Solving for *v*_1_, *v*_2_, *v*_3_ and *v*_4_, we get the left eigenvector *v* = (*v*_1_, *v*_2_, *v*_3_, *v*_4_) such that

$$v_{1}=0,\;v_{2}=0,\;v_{4}=0,\;v_{3}=v_{3}>0.$$

The eigenvectors *w* and *v* should satisfy the requirement *v* ⋅ *w* = 1, i.e.,

$$v_{3}w_{3}=1.$$

For finding the direction of the bifurcation, we need to determine the sign of bifurcation coefficients *a* and *b* which are given by

$a=\underset{k,i,j=1}{\sum^{3}}v_{k}w_{i}w_{j}\frac{\partial^{2}f_{k}}{\partial x_{i}\partial x_{j}}\left(E_{c}^{0},\beta_{c}^{*}\right)$
$b=\underset{k,i=1}{\sum^{3}}v_{k}w_{i}\frac{\partial^{2}f_{k}}{\partial x_{i}\partial \beta_{c}}\left(E_{c}^{0},\beta_{c}^{*}\right)$.

Since *v*_1_ = 0, *v*_2_ = 0, and *v*_4_ = 0, we do not need the derivatives of *f*_1_, *f*_2_ and *f*_4_. Thus, we need only the second order partial derivatives of *f*_3_ where

$$f_{3}=\left(1-\kappa \rho \right)\beta_{c}\frac{x_{1}x_{3}}{x_{1}+x_{2}+x_{3}+x_{4}}+\left(1-\varepsilon \right)\left(1-\kappa \rho \right)\beta_{c}\frac{x_{2}x_{3}}{x_{1}+x_{2}+x_{3}+x_{4}}-k_{0}x_{3}.$$

Therefore, from the second order partial derivatives of *f*_3_ at $\left(E_{c}^{0},\beta_{c}^{*}\right)$, the only ones that are nonzero are the following:

$$\frac{\partial^{2}f_{3}}{\partial x_{3}\partial x_{1}}\left(E_{c}^{0},\beta_{c}^{*}\right)=\frac{\left(1-\kappa \rho \right)\beta_{c}^{*}\nu \varepsilon \mu }{\Lambda \left(\nu +\mu \right)},\;\frac{\partial^{2}f_{3}}{\partial x_{3}\partial x_{2}}\left(E_{c}^{0},\beta_{c}^{*}\right)=-\frac{\left(1-\kappa \rho \right)\beta_{c}^{*}\varepsilon \mu^{2}}{\Lambda \left(\nu +\mu \right)},$$

$$\frac{\partial^{2}f_{3}}{\partial x_{3}^{2}}\left(E_{c}^{0},\beta_{c}^{*}\right)=-2\frac{\left(1-\kappa \rho \right)\beta_{c}^{*}\left(\mu +\left(1-\varepsilon \right)\nu \right)\mu }{\Lambda \left(\nu +\mu \right)},\;\text{and}\;\frac{\partial^{2}f_{3}}{\partial x_{4}\partial x_{3}}\left(E_{c}^{0},\beta_{c}^{*}\right)=-\frac{\left(1-\kappa \rho \right)\beta_{c}^{*}\left(\mu +\left(1-\varepsilon \right)\nu \right)\mu }{\Lambda \left(\nu +\mu \right)}.$$

Furthermore,

$$\frac{\partial^{2}f_{3}}{\partial x_{3}\partial \beta_{c}}\left(E_{c}^{0},\beta_{c}^{*}\right)=\frac{\left(1-\kappa \rho \right)\left[\mu +\left(1-\varepsilon \right)\nu \right]}{\nu +\mu }.$$

Now, using the above information, we obtain

$$a=v_{3}\left[2w_{1}w_{3}\frac{\partial^{2}f_{3}}{\partial x_{1}\partial x_{3}}\left(E_{c}^{0},\beta_{c}^{*}\right)+2w_{2}w_{3}\frac{\partial^{2}f_{3}}{\partial x_{2}\partial x_{3}}\left(E_{c}^{0},\beta_{c}^{*}\right)+w_{3}^{2}\frac{\partial^{2}f_{3}}{\partial x_{3}^{2}}\left(E_{c}^{0},\beta_{c}^{*}\right)+2w_{3}w_{4}\frac{\partial^{2}f_{3}}{\partial x_{3}\partial x_{4}}\left(E_{c}^{0},\beta_{c}^{*}\right)\right]=-2\frac{\mu k_{0}}{\Lambda \left(\mu +\left(1-\varepsilon \right)\nu \right)}\left[\varepsilon \left(\mu w_{2}-\nu w_{1}\right)+\left(\mu +\left(1-\varepsilon \right)\nu \right)\left(w_{3}+w_{4}\right)\right]v_{3}w_{3}<0,b=v_{3}\left(w_{3}\frac{\partial^{2}f_{3}}{\partial x_{3}\partial \beta_{c}}\right)=\frac{\left(1-\kappa \rho \right)\left[\mu +\left(1-\varepsilon \right)\nu \right]}{\nu +\mu }v_{3}w_{3}>0.$$

Since *a* < 0 and *b* > 0, by Theorem 4.1 stated in (Castillo-Chavez & Song, 2004), the COVID-19 sub-model (3) exhibit the phenomenon of forward bifurcation at *R*_*vC*_ = 1. Hence, the following result follows.

Lemma 5 *The unique endemic equilibrium (EE) of the COVID-19 sub-model* (3) *is locally asymptotically stable if* *R*_*vC*_ > 1*.*

The public health implication for the forward bifurcation is that the COVID-19 disease cannot invade the population for *R*_*vC*_ < 1. On the contrary, the disease persist in the community when *R*_*vC*_ exceeds unity.

### 3.2 TB sub-model

By setting *I*_*c*_(*t*) = *I*_*cL*_(*t*) = *I*_*tc*_(*t*) = *R*_*c*_(*t*) = 0 in model (1), we obtain the following TB-only model.

(14) $$\left\{\begin{array}{l} \begin{array}{cc} & \dot{S}=\Lambda -\left(\lambda_{t}+\mu \right)S,\\ & \dot{L}=\lambda_{t}\left(S+\varphi R_{t}\right)-\eta \lambda_{t}L-\left(\varphi_{t}+\mu \right)L,\\ & {\dot{I}}_{t}=\varphi_{t}L+\eta \lambda_{t}L-\left(r_{t}+\mu +\delta_{2}\right)I_{t},\\ & {\dot{R}}_{t}=r_{t}I_{t}-\left(\varphi \lambda_{t}+\mu \right)R_{t}, \end{array}\; \end{array}\right)$$

where $\lambda_{t}=\frac{\beta_{t}I_{t}}{N}$, and *N* = *S*(*t*) + *L*(*t*) + *I*_*t*_(*t*) + *R*_*t*_(*t*).

Analogously to Theorem 2, it can be shown that the region

$$\Omega_{T}=\left\{\left(S,L,I_{t},T\right)\in R_{+}^{4}:N\left(t\right)\leq \frac{\Lambda }{\mu }\right\}$$

is positively invariant for TB sub-model (14). Thus, we consider the dynamics of the TB sub-model (14) in Ω_*T*_.

#### 3.2.1 Disease-free equilibrium and basic reproduction number

System (14) always has the disease-free equilibrium

$$E_{t}^{0}=\left(S^{0},L^{0},I_{t}^{0},R_{t}^{0}\right)=\left(\frac{\Lambda }{\mu },0,0,0\right).$$

The basic reproduction number for system (14) is calculated in Appendix A and is given by

(15) $$R_{0T}=\left(\frac{\beta_{t}}{r_{t}+\mu +\delta_{2}}\right)\left(\frac{\varphi_{t}}{\varphi_{t}+\mu }\right).$$

This number is given by the product *β*_*t*_/(*r*_*t*_ + *μ* + *δ*_2_), that is, by the product of the average number of susceptibles infected by one infectious individual during his or her effective infectious period and *φ*_*t*_/(*φ*_*t*_ + *μ*), the fraction of the population which survives the latent period. Therefore, *R*_0*T*_ gives the number of secondary infectious cases produced by an infectious individual during his or her effective infectious period in a population of susceptible individuals.

Remark 1 The basic reproductive number *R*_0*T*_ does not depend on *η*. However, the number of endemic equilibria depends on the value of reinfection level *η*. The expression of *R*_0*T*_ here is essentially the same as that of TB sub-model without a exogenous reinfection term (*η* = 0). Here we show that as the value of *η* changes system (14) exhibits a backward bifurcation at *R*_0*T*_ = 1.

In the next section, we analyze the local asymptotic stability of the DFE $E_{t}^{0}$ of TB sub-model (14).

#### 3.2.2 Stability of the disease-free equilibrium

Lemma 6 ***(Local stability of DFE:)****The DFE*$E_{t}^{0}$*of the TB sub-model* (14) *is locally asymptotically stable if* *R*_0*T*_ < 1, *and unstable otherwise.*

*Proof*. The local stability of DFE $\left(E_{t}^{0}\right)$ of TB sub-model (14) is obtained using the sign of eigenvalues of the Jacobian matrix at $E_{t}^{0}$. The Jacobian matrix at $E_{t}^{0}$ of the TB sub-model (14) is given by

$$J_{E_{t}^{0}}=\left(\begin{array}{cccc} -\mu & 0 & -\beta_{t} & 0\\ 0 & -k_{1} & \beta_{t} & 0\\ 0 & \varphi_{t} & -k_{2} & 0\\ 0 & 0 & r_{t} & -\mu \end{array}\right),$$

where

$$k_{1}=\varphi_{t}+\mu \;\text{and}\;k_{2}=r_{t}+\mu +\delta_{2}.$$

It is obvious that *λ*_1_ = −*μ* = *λ*_4_ are the two negative eigenvalues of $J_{E_{t}^{0}}$. The remaining eigenvalues of $J_{E_{t}^{0}}$ are obtained from the block matrix

$$J_{1E_{t}^{0}}=\left(\begin{array}{cc} -k_{1} & \beta_{t}\\ \varphi_{t} & -k_{2} \end{array}\right).$$

The characteristic polynomial of $J_{1E_{t}^{0}}$ is given by

(16) $$P\left(\lambda \right)=\lambda^{2}+a_{1}\lambda +a_{2}=0,$$

where

*a*_1_ = *k*_1_ + *k*_2_,
$a_{2}=k_{1}k_{2}\left(1-R_{0_{t}}\right)$.

Because all model parameters are positive, *a*_1_ > 0 and *a*_2_ > 0 if and only if *R*_0*T*_ < 1. Therefore, by Routh-Hurwitz criterion, both roots of the characteristic polynomial (16) have negative real part, if *R*_0*T*_ < 1. Thus, all eigenvalues of the Jacobian matrix $J_{E_{t}^{0}}$ have a negative real part if *R*_0*T*_ < 1. If *R*_0*T*_ > 1, then *a*_3_ < 0 and the matrix $J_{E_{t}^{0}}$ has at least one eigenvalue with positive real part. Therefore, from this we conclude that the DFE $E_{t}^{0}$ of the TB sub-model system (14) is locally asymptotically stable if *R*_0*T*_ < 1 and unstable if *R*_0*T*_ > 1. □

The implication of Theorem 6 is that TB can be excluded from the community (for *R*_0*T*_ < 1) if the initial size of the model subpopulation lies in the attractive basin of $E_{t}^{0}$. Since some TB models can exhibit the phenomenon of backward bifurcation (see Sharomi, Podder, Gumel, and Song (2008) and the references therein), it is necessary to determine whether the current TB sub-model undergo this phenomenon.

#### 3.2.3 Existence of endemic equilibrium for TB sub-model

The endemic equilibrium point of the TB sub-model (14) is given by

$$E_{t}^{*}=\left(S^{*},L^{*},I_{t}^{*},R_{t}^{*}\right),$$

where

(17) $$\begin{array}{c} S^{*}=\frac{\Lambda }{\lambda_{t}^{*}+\mu },\\ L^{*}=\frac{\Lambda \lambda_{t}^{*}k_{2}\left(\varphi \lambda_{t}^{*}+\mu \right)}{\left(\lambda_{t}^{*}+\mu \right)\left[\left(\varphi_{t}+\eta \lambda_{t}^{*}\right)\left(\mu k_{2}+\varphi \lambda_{t}^{*}\left(\mu +\delta_{2}\right)\right)+\mu k_{2}\left(\varphi \lambda_{t}^{*}+\mu \right)\right]},\\ I_{t}^{*}=\frac{\Lambda \lambda_{t}^{*}\left(\varphi \lambda_{t}^{*}+\mu \right)\left(\varphi_{t}+\eta \lambda_{t}^{*}\right)}{\left(\lambda_{t}^{*}+\mu \right)\left[\left(\varphi_{t}+\eta \lambda_{t}^{*}\right)\left(\mu k_{2}+\varphi \lambda_{t}^{*}\left(\mu +\delta_{2}\right)\right)+\mu k_{2}\left(\varphi \lambda_{t}^{*}+\mu \right)\right]},\\ R_{t}^{*}=\frac{\Lambda \lambda_{t}^{*}r_{t}\left(\varphi_{t}+\eta \lambda_{t}^{*}\right)}{\left(\lambda_{t}^{*}+\mu \right)\left[\left(\varphi_{t}+\eta \lambda_{t}^{*}\right)\left(\mu k_{2}+\varphi \lambda_{t}^{*}\left(\mu +\delta_{2}\right)\right)+\mu k_{2}\left(\varphi \lambda_{t}^{*}+\mu \right)\right]}, \end{array}$$

such that

(18) $$\lambda_{t}^{*}=\frac{\beta_{t}I_{t}^{*}}{S^{*}+L^{*}+I_{t}^{*}+R_{t}^{*}},$$

while $\lambda_{t}^{*}$ is taken from the positive root of the following third degree polynomial

(19) $$P\left(\lambda_{t}^{*}\right)=A{\left(\lambda_{t}^{*}\right)}^{3}+B{\left(\lambda_{t}^{*}\right)}^{2}+C\lambda_{t}^{*}+D=0,$$

with

(20) $$\begin{array}{c} A=\varphi \eta \\ B=\varphi \eta \left(\mu +\delta_{2}\right)+\varphi \left(k_{2}+\varphi_{t}\right)+\eta \left(\mu +r_{t}\right)-\beta_{t}\varphi \eta \\ C=\varphi \varphi_{t}\left(\mu +\delta_{2}\right)+\mu k_{2}\left(\eta +\varphi \right)+\mu \left(k_{2}+\varphi_{t}\right)+r_{t}\varphi_{t}-\beta_{t}\left(\varphi \varphi_{t}+\mu \eta \right)\\ D=\mu k_{1}k_{2}\left(1-R_{0T}\right). \end{array}$$

Theorem 7 *TB sub-model* (14) *always has an endemic equilibrium whenever* *R*_0*T*_ > 1*.*

*Proof*. The proof follows easily from Theorem 2 in (Aldila & Angelina, 2021) and Theorem 3.3 in (Khajanchi, Das, & Kar, 2018). □

Theorem 7 implies that there always exists at least one EE if *R*_0*T*_ > 1. However, because $P\left(\lambda_{t}^{*}\right)$ is a third degree polynomial, we may have multiple endemic equilibria when *R*_0*T*_ > 1 or *R*_0*T*_ < 1. Thus, in light of the Descartes principles of signs, we examine the maximum number of positive roots of the polynomial (19). Table 3 provides a summary of the results. It can be observed that for *R*_0*T*_ < 1 the polynomial $P\left(\lambda_{t}^{*}\right)$ will either have zero or two positive roots. On the other hand, when *R*_0*T*_ > 1, we always have the possibility of having either one or three positive roots.

From Table 3, we can see that the TB sub-model (14) may have an EE even though *R*_0*T*_ < 1. Unfortunately, the results in Table 3 cannot provide a specific condition to guarantee the existence of the EE when *R*_0*T*_ < 1. Therefore, we continue the analysis by determining the possible conditions that guarantee the existence of EE when *R*_0*T*_ < 1, which is indicated by the negative sign of $\frac{\partial \lambda_{t}}{\partial R_{0T}}$ when *R*_0*T*_ = 1 and *λ*_*t*_ = 0. Furthermore, if it is satisfied we have two positive endemic equilibria if *R*_0*T*_ < 1.

**Table 3:** Number of possible positive real roots of polynomial (19).

Cases	A	B	C	D	Condition of *R*_0*T*_	Possible positive root
1	+	+	+	–	*R*_0*T*_ > 1	1
2	+	+	–	–	*R*_0*T*_ > 1	1
3	+	–	+	–	*R*_0*T*_ > 1	1 or 3
4	+	–	–	–	*R*_0*T*_ > 1	1
5	+	+	+	+	*R*_0*T*_ < 1	0
6	+	+	–	+	*R*_0*T*_ < 1	0 or 2
7	+	–	+	+	*R*_0*T*_ < 1	0 or 2
8	+	–	–	+	*R*_0*T*_ < 1	0 or 2

First, we make *A*, *B*, *C*, and *D* in $P\left(\lambda_{t}^{*}\right)$ to make it a function depending on *R*_0*T*_ by changing *β*_*t*_ as

$$\beta_{t}^{*}=\frac{R_{0T}k_{1}k_{2}}{\varphi_{t}}.$$

Substituting $\beta_{t}=\beta_{t}^{*}$ into *A*, *B*, *C*, and *D*, and taking the partial derivative of *λ*_*t*_ with respect to *R*_0*T*_ from $P\left(\lambda_{t}^{*}\right)$ (i.e., differentiating Equation (19) implicitly with respect to *R*_0*T*_) and evaluating it in *R*_0*T*_ = 1, *λ*_*t*_ = 0, give us:

$$\frac{\partial \lambda_{t}}{\partial R_{0T}}{|}_{\lambda_{t}=0,R_{0T}=1}=-\frac{\partial D/\partial R_{0T}}{C\left(R_{0T}\right)}{|}_{\lambda_{t}=0,R_{0T}=1},$$

where

$$\frac{\partial D}{\partial R_{0T}}{|}_{\lambda_{t}=0,R_{0T}=1}=-\mu k_{1}k_{2}.$$

Because $\frac{\partial D}{\partial R_{0T}}<0$, $\frac{\partial \lambda_{t}}{\partial R_{0T}}<0$ if and only if *C*(*R*_0*T*_) < 0, or equivalently,

$$\eta >\eta^{*}=\frac{\varphi_{t}\left[\mu \left(k_{2}+\varphi_{t}\right)+\varphi_{t}r_{t}\left(1-\varphi \right)\right]}{\mu^{2}k_{2}}.$$

So, we get the following result.

Theorem 8 *TB sub-model* (14) *has two endemic equilibriums in* Ω_*T*_ *when* *R*_0*T*_ < 1 *if*

(21) $$\eta >\eta^{*}=\frac{\varphi_{t}\left[\mu \left(k_{2}+\varphi_{t}\right)+\varphi_{t}r_{t}\left(1-\varphi \right)\right]}{\mu^{2}k_{2}}.$$

Based on Theorems 7 and 8, we propose the possibility of multiple EE points for *R*_0*T*_ < 1; even the DFE is locally asymptotically stable. Therefore, we need to understand these phenomena in more detail. The local stability of this EE is detailed in the next section.

#### 3.2.4 Existence of backward bifurcation

In this section, we employ the same strategy as in Section 3.1 to investigate direction of the bifurcation using the well-known Castillo-Chavez and Song bifurcation theorem (Castillo-Chavez & Song, 2004). To apply this theory, the following simplification and change of variables are made first of all.

Denoting $x={\left(x_{1},x_{2},x_{3},x_{4}\right)}^{T}={\left(S,L,I_{t},R_{t}\right)}^{T}$, the TB sub-model (14) can be rewritten in the form $\frac{dx}{dt}=f\left(x\right)$, where $f\left(x\right)=\left(f_{1}\left(x\right),f_{2}\left(x\right),f_{3}\left(x\right),f_{4}\left(x\right)\right)$ as follows

(22) $$\left\{\begin{array}{l} \begin{array}{cc} & \frac{dx_{1}}{dt}=f_{1}\left(x\right)=\Lambda -\left(\lambda_{t}+\mu \right)x_{1},\\ & \frac{dx_{2}}{dt}=f_{2}\left(x\right)=\lambda_{t}\left(x_{1}+\varphi x_{4}\right)-\eta \lambda_{t}x_{2}-\left(\varphi_{t}+\mu \right)x_{2},\\ & \frac{dx_{3}}{dt}=f_{3}\left(x\right)=\varphi_{t}x_{2}+\eta \lambda_{t}x_{2}-\left(r_{t}+\mu +\delta_{2}\right)x_{3},\\ & \frac{dx_{4}}{dt}=f_{4}\left(x\right)=r_{t}x_{3}-\left(\varphi \lambda_{t}+\mu \right)x_{4}, \end{array}\; \end{array}\right)$$

where $\lambda_{t}=\frac{\beta_{t}x_{3}}{x_{1}+x_{2}+x_{3}+x_{4}}$. Choosing $\beta_{t}^{*}$ as the bifurcation parameter and setting *R*_0*T*_ = 1, we get

$$\beta_{t}=\beta_{t}^{*}=\frac{\left(\varphi_{t}+\mu \right)\left(r_{t}+\mu +\delta_{2}\right)}{\varphi_{t}}.$$

The Jacobian matrix of the system (22) evaluated at $E_{t}^{0}$ and $\beta_{t}=\beta_{t}^{*}$ is given by

$$J_{\left(E_{t}^{0},\beta_{t}^{*}\right)}=\left(\begin{array}{cccc} -\mu & 0 & -\beta_{t}^{*} & 0\\ 0 & -\left(\varphi_{t}+\mu \right) & \beta_{t}^{*} & 0\\ 0 & \varphi_{t} & -\left(r_{t}+\mu +\delta_{2}\right) & 0\\ 0 & 0 & r_{t} & -\mu \end{array}\right).$$

Obviously, *λ*_1_ = −*μ* = *λ*_4_ are the two negative eigenvalues of $J_{\left(E_{t}^{0},\beta_{t}^{*}\right)}$ and the remaining eigenvalues are the roots of the characteristic equation

$$\lambda^{2}+\left[\left(\varphi_{t}+\mu \right)+\left(r_{t}+\delta_{2}+\mu \right)\right]\lambda =0,$$

which are *λ*_2_ = 0 and *λ*_3_ = −[(*φ*_*t*_ + *μ*) + (*r*_*t*_ + *δ*_2_ + *μ*)]. Thus, $J_{\left(E_{t}^{0},\beta_{t}^{*}\right)}$ has a simple zero eigenvalue and other eigenvalues have negative sign. Hence $E_{t}^{0}$ is a non-hyperbolic equilibrium, when $\beta_{t}=\beta_{t}^{*}$.

Next, we calculate the right and left eigenvectors associated to the zero eigenvalue.*Right eigenvector:* Let $w={\left(w_{1},w_{2},w_{3},w_{4}\right)}^{T}$ be the right eigenvector associated to the zero eigenvalue. i.e.,

$$J_{\left(E_{t}^{0},\beta_{t}^{*}\right)}\cdot w=0.$$

Solving for *w*_1_, *w*_2_, *w*_3_ and *w*_4_, we get the right eigenvector $w={\left(w_{1},w_{2},w_{3},w_{4}\right)}^{T}$ such that

$$w_{1}=-\frac{\left(\varphi_{t}+\mu \right)\left(r_{t}+\mu +\delta_{2}\right)}{\mu \varphi_{t}}w_{3},\;w_{2}=\frac{r_{t}+\mu +\delta_{2}}{\varphi_{t}}w_{3},\;w_{4}=\frac{r_{t}}{\mu }w_{3},\;w_{3}=w_{3}>0.$$

*Left eigenvector:* Let *v* = (*v*_1_, *v*_2_, *v*_3_, *v*_4_) be the left eigenvector associated to the zero eigenvalue. i.e.,

$$v\cdot J_{\left(E_{t}^{0},\beta_{t}^{*}\right)}=0.$$

Solving for *v*_1_, *v*_2_, *v*_3_ and *v*_4_, we get the left eigenvector *v* = (*v*_1_, *v*_2_, *v*_3_, *v*_4_) such that

$$v_{1}=0,\;v_{4}=0,\;v_{2}=\frac{\varphi_{t}}{\varphi_{t}+\mu }v_{3},\;v_{3}=v_{3}>0.$$

The eigenvectors *w* and *v* should satisfy the requirement *v* ⋅ *w* = 1, i.e.,

$$v_{3}w_{3}\left(1+\frac{r_{t}+\mu +\delta_{2}}{\varphi_{t}+\mu }\right)=1.$$

Next, to find the direction of the bifurcation, we need to determine the sign of bifurcation coefficients *a* and *b* defined as

$a=\underset{k,i,j=1}{\sum^{4}}v_{k}w_{i}w_{j}\frac{\partial^{2}f_{k}}{\partial x_{i}\partial x_{j}}\left(E_{t}^{0},\beta_{t}^{*}\right)$
$b=\underset{k,i=1}{\sum^{4}}v_{k}w_{i}\frac{\partial^{2}f_{k}}{\partial x_{i}\partial \beta_{t}}\left(E_{t}^{0},\beta_{t}^{*}\right)$.

Since *v*_1_ = *v*_4_ = 0, we do not need the derivatives of *f*_1_ and *f*_4_. Thus, we need only the second-order partial derivatives of *f*_2_ and *f*_3_ where

$$f_{2}=\frac{\beta_{t}x_{3}\left(x_{1}+\varphi x_{4}\right)}{x_{1}+x_{2}+x_{3}+x_{4}}-\frac{\eta \beta_{t}x_{3}x_{2}}{x_{1}+x_{2}+x_{3}+x_{4}}-\left(\varphi_{t}+\mu \right)x_{2}$$

and

$$f_{3}=\varphi_{t}x_{2}+\frac{\eta \beta_{t}x_{3}x_{2}}{x_{1}+x_{2}+x_{3}+x_{4}}-\left(r_{t}+\mu +\delta_{2}\right)x_{3}.$$

Therefore, the nonzero second-order partial derivatives of *f*_2_ and *f*_3_ at $\left(E_{t}^{0},\beta_{t}^{*}\right)$ are

$$\frac{\partial^{2}f_{2}}{\partial x_{3}\partial x_{2}}\left(E_{t}^{0},\beta_{t}^{*}\right)=\frac{\partial^{2}f_{2}}{\partial x_{2}\partial x_{3}}\left(E_{t}^{0},\beta_{t}^{*}\right)=\frac{-\beta_{t}^{*}\left(1+\eta \right)}{x_{1}^{0}},\;\frac{\partial^{2}f_{2}}{\partial x_{3}^{2}}\left(E_{t}^{0},\beta_{t}^{*}\right)=\frac{-2\beta_{t}^{*}}{x_{1}^{0}},$$

$$\frac{\partial^{2}f_{2}}{\partial x_{4}\partial x_{3}}\left(E_{t}^{0},\beta_{t}^{*}\right)=\frac{\partial^{2}f_{2}}{\partial x_{3}\partial x_{4}}\left(E_{t}^{0},\beta_{t}^{*}\right)=\frac{-\beta_{t}^{*}\left(1-\varphi \right)}{x_{1}^{0}},\;\frac{\partial^{2}f_{2}}{\partial x_{3}\partial \beta_{t}}\left(E_{t}^{0},\beta_{t}^{*}\right)=1,$$

and

$$\frac{\partial^{2}f_{3}}{\partial x_{3}\partial x_{2}}\left(E_{t}^{0},\beta_{t}^{*}\right)=\frac{\partial^{2}f_{3}}{\partial x_{2}\partial x_{3}}\left(E_{t}^{0},\beta_{t}^{*}\right)=\frac{\eta \beta_{t}^{*}}{x_{1}^{0}}.$$

Now, using the above information we obtain the bifurcation coefficients *a* and *b* as follows.

$$\begin{array}{ccc} a & =\underset{k,i,j=1}{\overset{4}{\sum }}v_{k}w_{i}w_{j}\frac{\partial^{2}f_{k}}{\partial x_{i}\partial x_{j}}\left(E_{t}^{0},\beta_{t}^{*}\right) & \\ & =v_{2}\left[2w_{2}w_{3}\frac{\partial^{2}f_{2}}{\partial x_{3}\partial x_{2}}\left(E_{t}^{0},\beta_{t}^{*}\right)+w_{3}^{2}\frac{\partial^{2}f_{2}}{\partial x_{3}^{2}}\left(E_{t}^{0},\beta_{t}^{*}\right)+2w_{3}w_{4}\frac{\partial^{2}f_{2}}{\partial x_{4}\partial x_{3}}\left(E_{t}^{0},\beta_{t}^{*}\right)\right]+v_{3}\left[2w_{2}w_{3}\frac{\partial^{2}f_{3}}{\partial x_{3}\partial x_{2}}\left(E_{t}^{0},\beta_{t}^{*}\right)\right] & \\ & =-2\frac{k_{2}}{\Lambda \varphi_{t}}\Upsilon v_{3}w_{3}^{2}+2\eta \frac{\mu^{2}k_{2}^{2}}{\Lambda \varphi_{t}^{2}}v_{3}w_{3}^{2}, & \end{array}$$

where *Υ* = *μ*(*k*_2_ + *φ*_*t*_) + *φ*_*t*_*r*_*t*_(1 − *φ*) > 0 and *k*_2_ = *r*_*t*_ + *μ* + *δ*_2_.

$$\begin{array}{ccc} b & =\underset{k,i=1}{\overset{4}{\sum }}v_{k}w_{i}\frac{\partial^{2}f_{k}}{\partial x_{i}\partial \beta_{t}}\left(E_{t}^{0},\beta_{t}^{*}\right) & \\ & =v_{2}w_{3}\frac{\partial^{2}f_{2}}{\partial x_{3}\partial \beta_{t}}\left(E_{t}^{0},\beta_{t}^{*}\right) & \\ & =\frac{\varphi_{t}}{\varphi_{t}+\mu }v_{3}w_{3}>0. & \end{array}$$

Following Theorem 4.1 in Castillo-Chavez and Song that the model (14) experiences the phenomena of backward bifurcation at *R*_0*T*_ = 1 whenever both *b* > 0 and

$$a=-2\frac{k_{2}}{\Lambda \varphi_{t}}\Upsilon v_{3}w_{3}^{2}+2\eta \frac{\mu^{2}k_{2}^{2}}{\Lambda \varphi_{t}^{2}}v_{3}w_{3}^{2}>0.$$

Hence, the following conclusion may be drawn.

Theorem 9 *The TB sub-model* (14) *undergoes backward bifurcation at* *R*_0*T*_ = 1 *if*

(23) $$\eta >\frac{\varphi_{t}}{\mu^{2}k_{2}}\Upsilon =\eta^{*}.$$

Note that the condition *η*∗ in equation (23) has the same condition as inequality (21). It is clear from Theorem 9 that the backward bifurcation phenomenon will not occur in the absence of re-infection (i.e., *η* = 0), because the right-hand side of the inequality in Theorem 9 is non-negative. To further confirm that there is no backward bifurcation in model (14) when no reinfection occurs (i.e., *η* = 0), one can check the global-asymptotic stability for the DFE.

From an epidemiological point of view, the occurrence of backward/subcritical bifurcation implies that having *R*_0*T*_ < 1, although necessary, is no longer sufficient for disease control.

Having analyzed the dynamics of the two sub-models, the full COVID-19-TB model (1) will now be analysed.

### 3.3 Analysis of the full TB-COVID-19 model

In this section, we analyze the full TB-COVID-19 co-infection model given by (1).

#### 3.3.1 Stability of the disease-free equilibrium

The disease-free equilibrium of the full TB-COVID-19 model (1) is given by

$$E^{0}=\left(S^{0},V^{0},I_{c}^{0},R_{c}^{0},L^{0},I_{t}^{0},R_{t}^{0},I_{cL}^{0},I_{tc}^{0}\right)=\left(\frac{\Lambda }{\nu +\mu },\frac{\Lambda \nu }{\mu \left(\nu +\mu \right)},0,0,0,0,0,0,0\right).$$

It is easy to show, using the next generation method (as in the previous sections), that the associated reproduction number for the full TB-COVID-19 model system (1) (denoted by *R*_0_) is given by

$$R_{0}=\max\left\{R_{vC},R_{0T}\right\},$$

where *R*_*vC*_ and *R*_0*T*_ are the basic reproduction numbers corresponding to COVID-19 only and TB-only submodels, given by Eqs. (4), (15), respectively. This implies that the dynamics of the TB-COVID-19 coinfection will be dominated by the disease with the bigger basic reproduction number.

Lemma 10 *The DFE of the TB-COVID-19 model* (1) *is locally asymptotically stable if the threshold parameter* *R*_0_ < 1*, and unstable if* *R*_0_ > 1*.*

*Proof*. The Jacobian matrix for the system (1) evaluated at the DFE *E*^0^ is given by

$$J_{E^{0}}=\left(\begin{array}{ccccccccc} -k_{1} & 0 & \frac{-\beta_{c}\left(1-\kappa \rho \right)\mu }{\nu +\mu } & 0 & 0 & \frac{-\beta_{t}\mu }{\nu +\mu } & 0 & 0 & 0\\ \nu & -\mu & \frac{-\beta_{c}\left(1-\kappa \rho \right)\left(1-\varepsilon \right)\nu }{\nu +\mu } & 0 & 0 & \frac{-\beta_{t}\nu }{\nu +\mu } & 0 & 0 & 0\\ 0 & 0 & \frac{\beta_{c}\left(1-\kappa \rho \right)\left[\mu +\left(1-\varepsilon \right)\nu \right]}{\nu +\mu }-k_{2} & 0 & 0 & 0 & 0 & 0 & \alpha_{c}\\ 0 & 0 & r_{c} & -\mu & 0 & 0 & 0 & 0 & 0\\ 0 & 0 & 0 & 0 & -k_{3} & \beta_{t} & 0 & \eta_{L} & 0\\ 0 & 0 & 0 & 0 & \varphi_{t} & -k_{4} & 0 & 0 & \theta \\ 0 & 0 & 0 & 0 & 0 & r_{t} & -\mu & 0 & 0\\ 0 & 0 & 0 & 0 & 0 & 0 & 0 & -k_{5} & 0\\ 0 & 0 & 0 & 0 & 0 & 0 & 0 & \eta_{c} & -k_{6} \end{array}\right),$$

where

(24) $$k_{1}=\nu +\mu ,\;k_{2}=r_{c}+\mu +\delta_{1},\;k_{3}=\varphi_{t}+\mu ,\;k_{4}=r_{t}+\mu +\delta_{2},\;k_{5}=\eta_{c}+\eta_{L}+\mu ,\;k_{6}=\alpha_{c}+\theta +\mu +\delta_{3}.$$

We can easily find the eigenvalues of $J_{E^{0}}$ from the following block matrices

$$J_{1E^{0}}=\left(\begin{array}{cc} -k_{1} & 0\\ \nu & -\mu \end{array}\right),\;J_{2E^{0}}=\left(\begin{array}{cc} \frac{\beta_{c}\left(1-\kappa \rho \right)\left[\mu +\left(1-\varepsilon \right)\nu \right]}{\nu +\mu }-k_{2} & 0\\ r_{c} & -\mu \end{array}\right),$$

$$J_{3E^{0}}=\left(\begin{array}{ccc} -k_{3} & \beta_{t} & 0\\ \varphi_{t} & -k_{4} & 0\\ 0 & r_{t} & -\mu \end{array}\right),\;J_{4E^{0}}=\left(\begin{array}{cc} -k_{5} & 0\\ \eta_{c} & -k_{6} \end{array}\right).$$

Obviously from the above block matrices, we obtained the eigenvalues *λ*_1_ = −*k*_1_, *λ*_2_ = −*μ*, *λ*_3_ = −*μ*, *λ*_4_ = *k*_2_(*R*_*vC*_ − 1), *λ*_7_ = −*μ*, *λ*_8_ = −*k*_5_ and *λ*_9_ = −*k*_6_. The remaining eigenvalues of $J_{E^{0}}$ are the roots of the characteristic polynomial

(25) $$\lambda^{2}+a_{1}\lambda +a_{2}=0,$$

where

*a*_1_ = *k*_1_ + *k*_4_
*a*_2_ = *k*_1_*k*_4_[1 − *R*_0*T*_].

The first three and the last three eigenvalues of $J_{E^{0}}$ have a negative real part. On the other hand, the fourth eigenvalue and the polynomial (25) will give eigenvalues with a negative real part, if $R_{0}=\max\left\{R_{0C},R_{0T}\right\}<1$. Hence, *E*^0^ is locally asymptotically stable if *R*_0_ < 1 and unstable otherwise. □

The full TB-COVID-19 model (1) will experience backward bifurcation under the same conditions as the TB sub-model (14), which may experience the phenomena of backward bifurcation (Mtisi, Rwezaura, & Tchuenche, 2009; Mukandavire, Gumel, Garira, & Tchuenche, 2009; Okosun & Makinde, 2013; Tchoumi et al., 2021). Applying the centre manifold theory described in (Castillo-Chavez & Song, 2004), the following result is established.

Theorem 11 *If the bifurcation parametersa* > 0 *and* *b* > 0*, the full model* (1) *will exhibit the phenomenon of backward bifurcation at* *R*_0_ = 1*.*

For the proof, one can follow the same approach as in Section 3.2.4. It should be noted that the occurrence of backward bifurcation prevents the model's equilibria from having global asymptotic stability. That is, both the DFE and endemic equilibria can only be locally asymptotically stable.

In the next section, we formulate and analyze the optimal control problem.

## 4 The optimal control model

To examine the possible effects of applying intervention strategies to slow the spread of COVID-19, TB, and their co-infection, we incorporate the time dependent controls in the model (1). We take into account the following model equations for the optimal control problem.

(26) $$\left\{\begin{array}{l} \begin{array}{cc} & \dot{S}=\Lambda -\left(\left(1-u_{1}\right)\lambda_{c}+\lambda_{t}+\left(\nu +u_{2}\right)+\mu \right)S,\\ & \dot{V}=\left(\nu +u_{2}\right)S-\left[\left(1-\varepsilon \right)\left(1-u_{1}\right)\lambda_{c}+\mu +\lambda_{t}\right]V,\\ & {\dot{I}}_{c}=\left(1-u_{1}\right)\lambda_{c}\left[S+\left(1-\varepsilon \right)V+R_{t}\right]+\left(\alpha_{c}+u_{3}\right)I_{tc}-\left(r_{c}+\omega \lambda_{t}+\mu +\delta_{1}\right)I_{c},\\ & \dot{R_{c}}=r_{c}I_{c}-\left(\lambda_{t}+\mu \right)R_{c},\\ & \dot{L}=\lambda_{t}\left[S+V+R_{c}+\varphi R_{t}\right]+\eta_{L}I_{cL}-\left(\eta \lambda_{t}+\varphi_{t}+\left(1-u_{1}\right)\lambda_{c}+\mu \right)L,\\ & {\dot{I}}_{t}=\left(\varphi_{t}+\eta \lambda_{t}\right)L+\theta I_{tc}-\left(\sigma \left(1-u_{1}\right)\lambda_{c}+\left(r_{t}+u_{3}\right)+\mu +\delta_{2}\right)I_{t},\\ & \dot{R_{t}}=\left(r_{t}+u_{3}\right)I_{t}-\left(\left(1-u_{1}\right)\lambda_{c}+\varphi \lambda_{t}+\mu \right)R_{t},\\ & {\dot{I}}_{cL}=\omega \lambda_{t}I_{c}+\left(1-u_{1}\right)\lambda_{c}L-\left(\eta_{c}+\eta_{L}+\mu \right)I_{cL},\\ & {\dot{I}}_{tc}=\sigma \left(1-u_{1}\right)\lambda_{c}I_{t}+\eta_{c}I_{cL}-\left(\left(\alpha_{c}+u_{3}\right)+\theta +\mu +\delta_{3}\right)I_{tc}, \end{array}\; \end{array}\right)$$

where

(i) *u*_1_(*t*) represents COVID-19 prevention strategies (such as face mask, hand washing, physical distancing),
(ii) *u*_2_(*t*) represents time-dependent COVID-19 vaccination rate,
(iii) *u*_3_(*t*) denotes TB treatment,

and *λ*_*c*_ and *λ*_*t*_ are as in Section 2 with initial condition given in (2).

The controls *u*_*i*_(⋅) are bounded between 0 and *u*_*i*__max_, with *u*_*i*__max_ < 1, *i* = 1, 2, 3. No additional preventative measures are taken to reduce either of diseases when the controls vanish. We assume that *u*_*i*__max_ is never equal to 1, since it makes the model more realistic from a medical point of view and thus we assumed *u*_*i*__max_ ≤ 1 − *δ*, *i* = 1, 2, 3, where *δ* ≪ 1 denotes a positive real number. We denote *u* = (*u*_1_, *u*_2_, *u*_3_) for our conveniences and later use.

Here, the target is to minimize disease burden and related costs. That is, to reduce the cost of COVID-19 prevention, the cost of COVID-19 vaccination, and the cost of TB treatment for active TB cases. Hence, we formulate the cost functional for the minimization problem as

(27) $$J\left(u\right)=\int_{0}^{t_{f}}\left(A_{1}I_{c}\left(t\right)+A_{2}I_{t}\left(t\right)+A_{3}I_{cL}\left(t\right)+A_{4}I_{tc}\left(t\right)+\frac{1}{2}\overset{3}{\underset{i=1}{\sum }}W_{i}u_{i}^{2}\right)dt\to \min$$

subject to (26) with its initial conditions. The positive coefficients *A*_1_, *A*_2_, …, *A*_4_ are the weight constants associated with COVID-19 infection, TB infection, COVID-19 and latent TB co-infection, and COVID-19 and active TB co-infection, respectively. The parameters *W*_1_, *W*_2_, and *W*_3_ represent the weight constants that measure the relative cost of the interventions connected to the control *u*_1_, *u*_2_, and *u*_3_, respectively. Larger the values of *W*_1_, *W*_2_ and *W*_3_, more expensive is the implementation cost for detection. The fixed constant *t*_*f*_ denotes the final intervention time.

Moreover, the terms *A*_1_*I*_*c*_(*t*), *A*_2_*I*_*t*_(*t*), *A*_3_*I*_*cL*_(*t*) and *A*_4_*I*_*tc*_(*t*) denote, respectively, the cost incurred due to COVID-19 infection, TB infection, COVID-19 and latent TB co-infection, and COVID-19 and active TB co-infection. We take a quadratic form for the cost of control as an approximation to the real nonlinear functional that depends on the assumption that the cost takes a nonlinear form (Kumar, Srivastava, Dong, & Takeuchi, 2020). As a result, we prevent cases of singular or bang-bang optimal control (Fleming & Rishel, 2012; Pontryagin, 1987).

Here, our goal is to find the optimal control $u^{*}=\left(u_{1}^{*},u_{2}^{*},u_{3}^{*}\right)$ of the control functions *u*(*t*) = (*u*_1_(*t*), *u*_2_(*t*), *u*_3_(*t*)) such that the associated state trajectories are the solution of the system (26) in the intervention time interval [0, *t*_*f*_] and minimize the cost functional provided in (27). More specifically,

(28) $$J\left(u^{*}\left(t\right)\right)=\min\left(J\left(u\right):u\in U\right),$$

subject to the state system (26), where

(29) $$U=\left\{u=\left(u_{1},u_{2},u_{3}\right):\;\text{are measurable and}\;\;0\leq u_{i}\left(t\right)\leq 1,\;i=1,2,3,\forall t\in \left[0,t_{f}\right]\right\}$$

is an admissible control set. The Pontryagin's Minimum Principle (Fleming & Rishel, 2012) helps to reduce problems (26), (27) and (29) to a problem of minimizing the Hamiltonian H given as:

(30) $$H=A_{1}I_{c}\left(t\right)+A_{2}I_{t}\left(t\right)+A_{3}I_{cL}\left(t\right)+A_{4}I_{tc}\left(t\right)+\frac{1}{2}\overset{3}{\underset{i=1}{\sum }}W_{i}u_{i}^{2}+\overset{9}{\underset{i=1}{\sum }}\xi_{i}\left(t\right)g_{i}\left(t,S,V,I_{c},R_{c},L,I_{t},R_{t},I_{cL},I_{tc},u\right)$$

where *g*_*i*_ represents the set of right-hand side functions in (26) and $\xi :\left[0,t_{f}\right]\to R^{9}$, *ξ* = (*ξ*_1_(*t*), *ξ*_2_(*t*), …*ξ*_9_(*t*)) is a nontrivial absolutely continuous mapping called adjoint vector. In the following, the existence and characterization of the optimal controls are detailed.

### 4.1 Existence of the optimal control

In this subsection, the existence of optimal controls that minimize the cost function in the finite time is discussed. In this case, the boundedness of solutions to control-induced system (26) is very critical in determining the existence and uniqueness of optimal control (Abraha, Al Basir, Obsu, & Torres, 2021).

Theorem 12 *Given the objective functional* (27) *subjected to the control-induced initial value problem* (26)*, there exists optimal control triple* $u^{*}=\left(u_{1}^{*},u_{2}^{*},u_{3}^{*}\right)$ *in* U *and corresponding state solutions (S*∗*,* *V*∗*,* $I_{c}^{*}$*,* $R_{c}^{*}$*,* *L*∗*,* $I_{t}^{*}$*,* $R_{t}^{*}$*,* $I_{cL}^{*}$*,* $I_{tc}^{*}$*) such that*

$$J\left(u^{*}\right)=\underset{U}{\min\;}J\left(u\right).$$

*Proof*. For the existence of the given optimal control *u*∗, we need to verify the following conditions given by Fleming and Rishel (2012).

(i) The set of solutions to system (26) with associated control functions in (29) is nonempty.
(ii) The control admissible set U (29) is convex and closed.
(iii) Each right-hand side of the state system (26) is continuous, bounded by a sum of the bounded control and the state.
(iv) The state system can be written as a linear function of control variables *u* = (*u*_1_, *u*_2_, *u*_3_), with the coefficients as functions of time and state variables.
(v) The integrand *F*(*t*, *S*, *V*, *I*_*c*_, *R*_*c*_, *L*, *I*_*t*_, *R*_*t*_, *I*_*cL*_, *I*_*tc*_, *u*) in equation (27) is convex with respect to control variables.
(vi) There exist positive constants *c*_1_, *c*_2_, *c*_3_, *c*_4_ and a constant *χ* > 1 such that

$$F\left(t,S,V,I_{c},R_{c},L,I_{t},R_{t},I_{cL},I_{tc},u\right)\geq c_{2}|u_{1}{|}^{\chi }+c_{3}|u_{2}{|}^{\chi }+c_{4}|u_{3}{|}^{\chi }-c_{1}$$

The state variables in the proposed model are continuously differentiable. Besides, it is proved in Theorem 2 that the solutions of the state system are continuous and bounded. Furthermore, the right-hand side functions of model (26) satisfy the Lipschitz condition with respect to the state variables, which is follows from the boundedness of the partial derivatives with respect to state variables in the state system. Therefore, the result follows from Picard-Lindel$\ddot{o}$f's existence theorem (Coddington & Levinson, 1955). Being a quadratic function of *u* = (*u*_1_, *u*_2_, *u*_3_), the integrand in equation (27) is convex on U.

Furthermore, for proving the condition in (vi), it can be seen that, $W_{3}u_{3}^{2}\leq W_{3}$ as *W*_3_ ∈ [0, 1], that is, $\frac{W_{3}u_{3}^{2}}{2}\leq \frac{W_{3}}{2}$. Thus,

$$\begin{array}{ccc} F\left(t,X,u\right)>\frac{W_{1}}{2}u_{1}^{2}+\frac{W_{2}}{2}u_{2}^{2}+\frac{W_{3}}{2}u_{3}^{2}\geq \\ \geq =\frac{W_{1}}{2}u_{1}^{2}+\frac{W_{2}}{2}u_{2}^{2}+\frac{W_{3}}{2}u_{3}^{2}-\frac{W_{3}}{2}\geq \\ \min\left(\frac{W_{1}}{2},\frac{W_{2}}{2},\frac{W_{3}}{2}\right)\left(u_{1}^{2}+u_{2}^{2}+u_{3}^{2}\right)-\frac{W_{3}}{2}=\\ & \min\left(\frac{W_{1}}{2},\frac{W_{2}}{2},\frac{W_{3}}{2}\right)|u_{1},u_{2},u_{3}{|}^{2}-\frac{W_{3}}{2}. & \end{array}$$

Choosing $c=\min\left(\frac{W_{1}}{2},\frac{W_{2}}{2},\frac{W_{3}}{2}\right)$ and $c_{1}=\frac{W_{3}}{2}$, we get *F*(*t*, *X*, *u*) ≥ *c*|*u*_1_, *u*_2_, *u*_3_|^*χ*^ − *c*_1_ with *χ* > 1. This completes the proof. □

### 4.2 Characterization of optimal control solutions

The Pontryagin's Maximum Principle (PMP) introduced in (Fleming & Rishel, 2012) provides the necessary optimality condition as stated in the following theorem.

Theorem 13 *Letu*∗ *be the optimal solution to* (26)*,*(27)*–*(29) *and (S*∗*,* *V*∗*,* $I_{c}^{*}$*,* $R_{c}^{*}$*,* *L*∗*,* $I_{t}^{*}$*,* $R_{t}^{*}$*,* $I_{cL}^{*}$*,* $I_{tc}^{*}$*) be the associated optimal state. Then there exist adjoint functions* *ξ*_1_, *ξ*_2_, *ξ*_3_, …, *ξ*_9_ *that satisfy the adjoint system*

$$\begin{array}{cc} \frac{d\xi_{1}}{dt}=\left(1-u_{1}\right)\frac{\lambda_{c}}{N}\left[\left(\xi_{3}-\xi_{1}\right)S+\left(\xi_{3}-\xi_{2}\right)\left(1-\varepsilon \right)V+\left(\xi_{3}-\xi_{7}\right)R_{t}+\left(\xi_{8}-\xi_{5}\right)L+\left(\xi_{9}-\xi_{6}\right)\sigma I_{t}\right]\\ & +\frac{\lambda_{t}}{N}\left[\left(\xi_{5}-\xi_{1}\right)S+\left(\xi_{5}-\xi_{2}\right)V+\left(\xi_{5}-\xi_{4}\right)R_{c}+\left(\xi_{5}-\xi_{7}\right)\varphi R_{t}+\left(\xi_{8}-\xi_{3}\right)\omega I_{c}+\left(\xi_{6}-\xi_{5}\right)\eta L\right]\\ & +\left(1-u_{1}\right)\left(\xi_{1}-\xi_{3}\right)\lambda_{c}+\left(\xi_{1}-\xi_{5}\right)\lambda_{t}+\left(\nu +u_{2}\right)\left(\xi_{1}-\xi_{2}\right)+\mu \xi_{1},\\ \frac{d\xi_{2}}{dt} & =\left(1-u_{1}\right)\frac{\lambda_{c}}{N}\left[\left(\xi_{3}-\xi_{1}\right)S+\left(\xi_{3}-\xi_{2}\right)\left(1-\varepsilon \right)V+\left(\xi_{3}-\xi_{7}\right)R_{t}+\left(\xi_{8}-\xi_{5}\right)L+\left(\xi_{9}-\xi_{6}\right)\sigma I_{t}\right]\\ & +\frac{\lambda_{t}}{N}\left[\left(\xi_{5}-\xi_{1}\right)S+\left(\xi_{5}-\xi_{2}\right)V+\left(\xi_{5}-\xi_{4}\right)R_{c}+\left(\xi_{5}-\xi_{7}\right)\varphi R_{t}+\left(\xi_{8}-\xi_{3}\right)\omega I_{c}+\left(\xi_{6}-\xi_{5}\right)\eta L\right]\\ & +\left(1-u_{1}\right)\left(1-\varepsilon \right)\left(\xi_{2}-\xi_{3}\right)\lambda_{c}+\left(\xi_{2}-\xi_{5}\right)\lambda_{t}+\mu \xi_{2},\\ \frac{d\xi_{3}}{dt} & =-A_{1}+\left(1-u_{1}\right)\frac{\lambda_{c}}{N}\left[\left(\xi_{3}-\xi_{1}\right)S+\left(\xi_{3}-\xi_{2}\right)\left(1-\varepsilon \right)V+\left(\xi_{3}-\xi_{7}\right)R_{t}+\left(\xi_{8}-\xi_{5}\right)L+\left(\xi_{9}-\xi_{6}\right)\sigma I_{t}\right]\\ & +\frac{\lambda_{t}}{N}\left[\left(\xi_{5}-\xi_{1}\right)S+\left(\xi_{5}-\xi_{2}\right)V+\left(\xi_{5}-\xi_{4}\right)R_{c}+\left(\xi_{5}-\xi_{7}\right)\varphi R_{t}+\left(\xi_{8}-\xi_{3}\right)\omega I_{c}+\left(\xi_{6}-\xi_{5}\right)\eta L\right]\\ & +\left(1-u_{1}\right)\left(1-\kappa \rho \right)\frac{\beta_{c}}{N}\left[\left(\xi_{1}-\xi_{3}\right)S+\left(\xi_{2}-\xi_{3}\right)\left(1-\varepsilon \right)V+\left(\xi_{7}-\xi_{3}\right)R_{t}+\left(\xi_{5}-\xi_{8}\right)L+\left(\xi_{6}-\xi_{9}\right)\sigma I_{t}\right]\\ & +\left(\xi_{3}-\xi_{8}\right)\frac{\omega }{N}+\left(r_{c}+\mu +\delta_{1}\right)\xi_{3}-r_{c}\xi_{4},\\ \frac{d\xi_{4}}{dt} & =\left(1-u_{1}\right)\frac{\lambda_{c}}{N}\left[\left(\xi_{3}-\xi_{1}\right)S+\left(\xi_{3}-\xi_{2}\right)\left(1-\varepsilon \right)V+\left(\xi_{3}-\xi_{7}\right)R_{t}+\left(\xi_{8}-\xi_{5}\right)L+\left(\xi_{9}-\xi_{6}\right)\sigma I_{t}\right]\\ & +\frac{\lambda_{t}}{N}\left[\left(\xi_{5}-\xi_{1}\right)S+\left(\xi_{5}-\xi_{2}\right)V+\left(\xi_{5}-\xi_{4}\right)R_{c}+\left(\xi_{5}-\xi_{7}\right)\varphi R_{t}+\left(\xi_{8}-\xi_{3}\right)\omega I_{c}+\left(\xi_{6}-\xi_{5}\right)\eta L\right]\\ & +\left(\xi_{4}-\xi_{5}\right)\lambda_{t}+\mu \xi_{4},\\ \frac{d\xi_{5}}{dt} & =\left(1-u_{1}\right)\frac{\lambda_{c}}{N}\left[\left(\xi_{3}-\xi_{1}\right)S+\left(\xi_{3}-\xi_{2}\right)\left(1-\varepsilon \right)V+\left(\xi_{3}-\xi_{7}\right)R_{t}+\left(\xi_{8}-\xi_{5}\right)L+\left(\xi_{9}-\xi_{6}\right)\sigma I_{t}\right]\\ & +\frac{\lambda_{t}}{N}\left[\left(\xi_{5}-\xi_{1}\right)S+\left(\xi_{5}-\xi_{2}\right)V+\left(\xi_{5}-\xi_{4}\right)R_{c}+\left(\xi_{5}-\xi_{7}\right)\varphi R_{t}+\left(\xi_{8}-\xi_{3}\right)\omega I_{c}+\left(\xi_{6}-\xi_{5}\right)\eta L\right]\\ & +\left(1-u_{1}\right)\left(\xi_{5}-\xi_{8}\right)\lambda_{c}+\left(\xi_{5}-\xi_{6}\right)\eta \lambda_{t}+\left(\varphi_{t}+\mu \right)\xi_{5}-\varphi_{t}\xi_{6},\\ \frac{d\xi_{6}}{dt} & =-A_{2}+\left(1-u_{1}\right)\frac{\lambda_{c}}{N}\left[\left(\xi_{3}-\xi_{1}\right)S+\left(\xi_{3}-\xi_{2}\right)\left(1-\varepsilon \right)V+\left(\xi_{3}-\xi_{7}\right)R_{t}+\left(\xi_{8}-\xi_{5}\right)L+\left(\xi_{9}-\xi_{6}\right)\sigma I_{t}\right]\\ & +\frac{\lambda_{t}}{N}\left[\left(\xi_{5}-\xi_{1}\right)S+\left(\xi_{5}-\xi_{2}\right)V+\left(\xi_{5}-\xi_{4}\right)R_{c}+\left(\xi_{5}-\xi_{7}\right)\varphi R_{t}+\left(\xi_{8}-\xi_{3}\right)\omega I_{c}+\left(\xi_{6}-\xi_{5}\right)\eta L\right]\\ & +\frac{\beta_{t}}{N}\left[\left(\xi_{1}-\xi_{5}\right)S+\left(\xi_{2}-\xi_{5}\right)V+\left(\xi_{4}-\xi_{5}\right)R_{c}+\left(\xi_{7}-\xi_{5}\right)\varphi R_{t}+\left(\xi_{3}-\xi_{8}\right)\omega I_{c}+\left(\xi_{5}-\xi_{6}\right)\eta L\right]\\ & +\left(1-u_{1}\right)\left(\xi_{6}-\xi_{9}\right)\sigma \lambda_{c}+\left(r_{t}+u_{3}+\mu +\delta_{2}\right)\xi_{6}-\left(r_{t}+u_{3}\right)\xi_{7},\\ \frac{d\xi_{7}}{dt} & =\left(1-u_{1}\right)\frac{\lambda_{c}}{N}\left[\left(\xi_{3}-\xi_{1}\right)S+\left(\xi_{3}-\xi_{2}\right)\left(1-\varepsilon \right)V+\left(\xi_{3}-\xi_{7}\right)R_{t}+\left(\xi_{8}-\xi_{5}\right)L+\left(\xi_{9}-\xi_{6}\right)\sigma I_{t}\right]\\ & +\frac{\lambda_{t}}{N}\left[\left(\xi_{5}-\xi_{1}\right)S+\left(\xi_{5}-\xi_{2}\right)V+\left(\xi_{5}-\xi_{4}\right)R_{c}+\left(\xi_{5}-\xi_{7}\right)\varphi R_{t}+\left(\xi_{8}-\xi_{3}\right)\omega I_{c}+\left(\xi_{6}-\xi_{5}\right)\eta L\right]\\ & +\left(1-u_{1}\right)\left(\xi_{7}-\xi_{3}\right)\lambda_{c}+\left(\xi_{7}-\xi_{5}\right)\varphi \lambda_{t}+\mu \xi_{7},\\ \frac{d\xi_{8}}{dt} & =-A_{3}+\left(1-u_{1}\right)\frac{\lambda_{c}}{N}\left[\left(\xi_{3}-\xi_{1}\right)S+\left(\xi_{3}-\xi_{2}\right)\left(1-\varepsilon \right)V+\left(\xi_{3}-\xi_{7}\right)R_{t}+\left(\xi_{8}-\xi_{5}\right)L+\left(\xi_{9}-\xi_{6}\right)\sigma I_{t}\right]\\ & +\frac{\lambda_{t}}{N}\left[\left(\xi_{5}-\xi_{1}\right)S+\left(\xi_{5}-\xi_{2}\right)V+\left(\xi_{5}-\xi_{4}\right)R_{c}+\left(\xi_{5}-\xi_{7}\right)\varphi R_{t}+\left(\xi_{8}-\xi_{3}\right)\omega I_{c}+\left(\xi_{6}-\xi_{5}\right)\eta L\right]\\ & +\left(1-u_{1}\right)\left(1-\kappa \rho \right)\tau \frac{\beta_{c}}{N}\left[\left(\xi_{1}-\xi_{3}\right)S+\left(\xi_{2}-\xi_{3}\right)\left(1-\varepsilon \right)V+\left(\xi_{7}-\xi_{3}\right)R_{t}+\left(\xi_{5}-\xi_{8}\right)L+\left(\xi_{6}-\xi_{9}\right)\sigma I_{t}\right]\\ & +\left(\eta_{c}+\eta_{L}+\mu \right)\xi_{8}-\eta_{L}\xi_{5}-\eta_{c}\xi_{9}, \end{array}$$

(31) $$\begin{array}{cc} \frac{d\xi_{9}}{dt} & =-A_{4}+\left(1-u_{1}\right)\frac{\lambda_{c}}{N}\left[\left(\xi_{3}-\xi_{1}\right)S+\left(\xi_{3}-\xi_{2}\right)\left(1-\varepsilon \right)V+\left(\xi_{3}-\xi_{7}\right)R_{t}+\left(\xi_{8}-\xi_{5}\right)L+\left(\xi_{9}-\xi_{6}\right)\sigma I_{t}\right]\\ & +\frac{\lambda_{t}}{N}\left[\left(\xi_{5}-\xi_{1}\right)S+\left(\xi_{5}-\xi_{2}\right)V+\left(\xi_{5}-\xi_{4}\right)R_{c}+\left(\xi_{5}-\xi_{7}\right)\varphi R_{t}+\left(\xi_{8}-\xi_{3}\right)\omega I_{c}+\left(\xi_{6}-\xi_{5}\right)\eta L\right]\\ & +\left(1-u_{1}\right)\left(1-\kappa \rho \right)\tau \frac{\beta_{c}}{N}\left[\left(\xi_{1}-\xi_{3}\right)S+\left(\xi_{2}-\xi_{3}\right)\left(1-\varepsilon \right)V+\left(\xi_{7}-\xi_{3}\right)R_{t}+\left(\xi_{5}-\xi_{8}\right)L+\left(\xi_{6}-\xi_{9}\right)\sigma I_{t}\right]\\ & +\frac{\beta_{t}}{N}\left[\left(\xi_{1}-\xi_{5}\right)S+\left(\xi_{2}-\xi_{5}\right)V+\left(\xi_{4}-\xi_{5}\right)R_{c}+\left(\xi_{7}-\xi_{5}\right)\varphi R_{t}+\left(\xi_{3}-\xi_{8}\right)\omega I_{c}+\left(\xi_{5}-\xi_{6}\right)\eta L\right]\\ & +\left(\alpha_{c}+u_{3}+\theta +\mu +\delta_{3}\right)\xi_{9}-\theta \xi_{6}-\left(\alpha_{c}+u_{3}\right)\xi_{3}, \end{array}$$

Subjected to transversality conditions

$$\xi_{i}\left(t_{f}\right)=0,\;for\;i=1,2,\ldots ,9.$$

*Furthermore*, *the optimal control*$u^{*}=\left(u_{1}^{*},u_{2}^{*},u_{3}^{*}\right)$*is characterized by*

(32) $$\begin{array}{cc} u_{1}^{*}\left(t\right) & =\min\left\{\max\left\{0,\frac{\left(1-\kappa \rho \right)\beta_{c}\left(I_{c}+\tau \left(I_{tc}+I_{cL}\right)\right)\left[\left(\xi_{3}-\xi_{1}\right)S+\left(\xi_{3}-\xi_{2}\right)\left(1-\varepsilon \right)V+\left(\xi_{3}-\xi_{7}\right)R_{t}+\left(\xi_{8}-\xi_{5}\right)L+\left(\xi_{9}-\xi_{6}\right)\sigma I_{t}\right]}{W_{1}N}\right\},1\right\},\\ & u_{2}^{*}\left(t\right)=\min\left\{\max\left\{0,\frac{\left(\xi_{1}-\xi_{2}\right)S}{W_{2}}\right\},1\right\},\\ & u_{3}^{*}\left(t\right)=\min\left\{\max\left\{0,\frac{\left(\xi_{6}-\xi_{7}\right)I_{t}+\left(\xi_{9}-\xi_{3}\right)I_{tc}}{W_{3}}\right\},1\right\}. \end{array}$$

*Proof*. The result follows directly from the PMP, which states that the optimal control problem's solution fulfills the adjoint equations and transversality requirements

$$\begin{array}{cc} & \frac{d\xi_{1}}{dt}=-\frac{\partial H}{\partial S},\;\;\;\frac{d\xi_{2}}{dt}=-\frac{\partial H}{\partial V},\;\;\;\frac{d\xi_{3}}{dt}=-\frac{\partial H}{\partial I_{c}},\;\;\;\frac{d\xi_{4}}{dt}=-\frac{\partial H}{\partial R_{c}},\;\;\;\frac{d\xi_{5}}{dt}=-\frac{\partial H}{\partial L},\\ & \frac{d\xi_{6}}{dt}=-\frac{\partial H}{\partial I_{t}},\;\;\;\frac{d\xi_{7}}{dt}=-\frac{\partial H}{\partial R_{t}},\;\;\;\frac{d\xi_{8}}{dt}=-\frac{\partial H}{\partial I_{cL}},\;\;\;\frac{d\xi_{9}}{dt}=-\frac{\partial H}{\partial I_{tc}}, \end{array}$$

with *ξ*_*i*_(*t*_*f*_) = 0, for *i* = 1, 2, 3, …, 9. Moreover, the convexity of the objective functional in (27) w.r.t *u* guarantees the existence of an optimal control (Fleming & Rishel, 2012). Thus, from the optimality conditions

$$\frac{\partial H}{\partial u_{i}}=0,\;i=1,2,3,$$

we obtain the optimal control *u*∗ explicitly in terms of state and adjoint variables as follows.

(33) $$\begin{array}{cc} u_{1}^{*}\left(t\right) & =\frac{\left(1-\kappa \rho \right)\beta_{c}\left(I_{c}+\tau \left(I_{tc}+I_{cL}\right)\right)\left[\left(\xi_{3}-\xi_{1}\right)S+\left(\xi_{3}-\xi_{2}\right)\left(1-\varepsilon \right)V+\left(\xi_{3}-\xi_{7}\right)R_{t}+\left(\xi_{8}-\xi_{5}\right)L+\left(\xi_{9}-\xi_{6}\right)\sigma I_{t}\right]}{W_{1}N},\\ u_{2}^{*}\left(t\right) & =\frac{\left(\xi_{1}-\xi_{2}\right)S}{W_{2}},\\ u_{3}^{*}\left(t\right) & =\frac{\left(\xi_{6}-\xi_{7}\right)I_{t}+\left(\xi_{9}-\xi_{3}\right)I_{tc}}{W_{3}}. \end{array}$$

From the boundedness of $u_{i}^{*}\left(t\right)$ on (0, 1) and the minimality condition, we have:

$$\;\;\;u_{1}^{*}=\left\{\begin{array}{ll} 0,\; & \text{if }\frac{\partial H}{\partial u_{1}}>0,\\ \frac{\left(1-\kappa \rho \right)\beta_{c}\left(I_{c}+\tau \left(I_{tc}+I_{cL}\right)\right)\left[\left(\xi_{3}-\xi_{1}\right)S+\left(\xi_{3}-\xi_{2}\right)\left(1-\varepsilon \right)V+\left(\xi_{3}-\xi_{7}\right)R_{t}+\left(\xi_{8}-\xi_{5}\right)L+\left(\xi_{9}-\xi_{6}\right)\sigma I_{t}\right]}{W_{2}N},\; & \text{if }\frac{\partial H}{\partial u_{1}}=0,\\ 1,\; & \text{if }\frac{\partial H}{\partial u_{1}}<0. \end{array}\right)$$

$$u_{2}^{*}\left(t\right)=\left\{\begin{array}{ll} 0,\; & \text{if }\frac{\partial H}{\partial u_{2}}>0,\\ \frac{\left(\xi_{1}-\xi_{2}\right)S}{W_{2}},\; & \text{if }\frac{\partial H}{\partial u_{2}}=0,\\ 1,\; & \text{if }\frac{\partial H}{\partial u_{2}}<0. \end{array}\right)\;\;u_{3}^{*}=\left\{\begin{array}{ll} 0,\; & \text{if }\frac{\partial H}{\partial u_{3}}>0,\\ \frac{\left(\xi_{6}-\xi_{7}\right)I_{t}+\left(\xi_{9}-\xi_{3}\right)I_{tc}}{W_{3}},\; & \text{if }\frac{\partial H}{\partial u_{3}}=0,\\ 1,\; & \text{if }\frac{\partial H}{\partial u_{3}}<0. \end{array}\right)$$

Concluding that the optimal control functions $u_{i}^{*}\left(t\right)$ can be written in compact form as in (32). □

To verify that the optimal control is indeed a minimizer, we need to compute the Hessian matrix of the Hamiltonian H function and check its positive definiteness. Indeed, the Hessian matrix for H w.r.t the control *u* is given by

$$\frac{\partial^{2}H}{\partial u^{2}}=\left[\begin{array}{ccc} W_{1} & 0 & 0\\ 0 & W_{2} & 0\\ 0 & 0 & W_{3} \end{array}\right].$$

Clearly, this matrix is a positive definite due to the positive weights *W*_1_,*W*_2_ and *W*_3_. This confirms that the corresponding Hamiltonian function H is convex, and as a result, the optimal control, *u* = (*u*_1_, *u*_2_, *u*_3_), is a minimizer (Abraha et al., 2021).

The proposed control induced system (26) is an initial value problem while the adjoint system (31) subject to the transversality conditions *ξ*_*i*_(*t*_*f*_) = 0, *i* = 1, …, 9 is a terminal value problem. Therefore, the boundary value problem given by Theorem 13 need to be solved numerically using forward iteration in the state system, and the adjoint system (31) is integrated through backward iteration (Campos, Silva, & Torres, 2019; Kumar et al., 2020; Lenhart & Workman, 2007; Silva & Torres, 2013).

## 5 Simulations of optimal control model

In this section, several numerical simulations of model (26) is performed to support the analytical results. To obtain the optimal solution to the control induced model (26), we apply the forward-backward sweep method presented in (Lenhart & Workman, 2007). This iterative method consists in solving the system of equations in (26) with the guess for the controls over the time interval [0, *t*_*f*_] using a forward fourth-order Runge-Kutta scheme and the transversality conditions. Then, the adjoint system is solved by applying a backward fourth-order Runge-Kutta scheme to the current iteration solution of the system of equation (26) (For details see references (Campos et al., 2019; Lenhart & Workman, 2007)). The controls are updated by means of a convex combination of the previous controls and the values in the characterization process (32). To repeat the solutions of state variables in (26) and adjoint systems, the updated controls are used. This situation is continued until the values of unknowns at the previous iteration and at the present iteration are close enough.

To assess the cost of each applied strategy, the following weight constants are presumptions.

$$A_{1}=A_{2}=A_{3}=10,\;A_{4}=100,\;W_{1}=100,\;W_{2}=50,\;W_{3}=1000.$$

Also, the cost of TB treatment is assumed to be higher than the cost of implementing COVID-19 prevention control (face-mask usage and vaccination).

### 5.1 Initial conditions

In this subsection, we define the initial conditions that correspond to the number of individuals in each class in order to solve model (1). In this regard, the initial total population is assumed to be *N*(0) = 114, 963, 588 (Kifle & Obsu, 2022). Further, we assume the initial conditions for the state variables used in model simulation as follows: the total number of fully vaccinated individuals against COVID-19 was *V*(0) = 400, 000, *L*(0) = 100, 000, *I*_*t*_(0) = 30, 000, *I*_*c*_(0) = 280, 565, *I*_*cL*_(0) = 50, 000, *I*_*tc*_(0) = 5, 000, *R*_*c*_(0) = 263, 587 and *R*_*t*_(0) = 7000. Thus, the initial susceptible population is determined as *S*(0) = *N*(0) − [*V*(0) + *I*_*c*_(0) + *R*_*c*_(0) + *L*(0) + *I*_*t*_(0) + *R*_*t*_(0) + *I*_*cL*_(0) + *I*_*tc*_(0)].

**Fig. 2:**
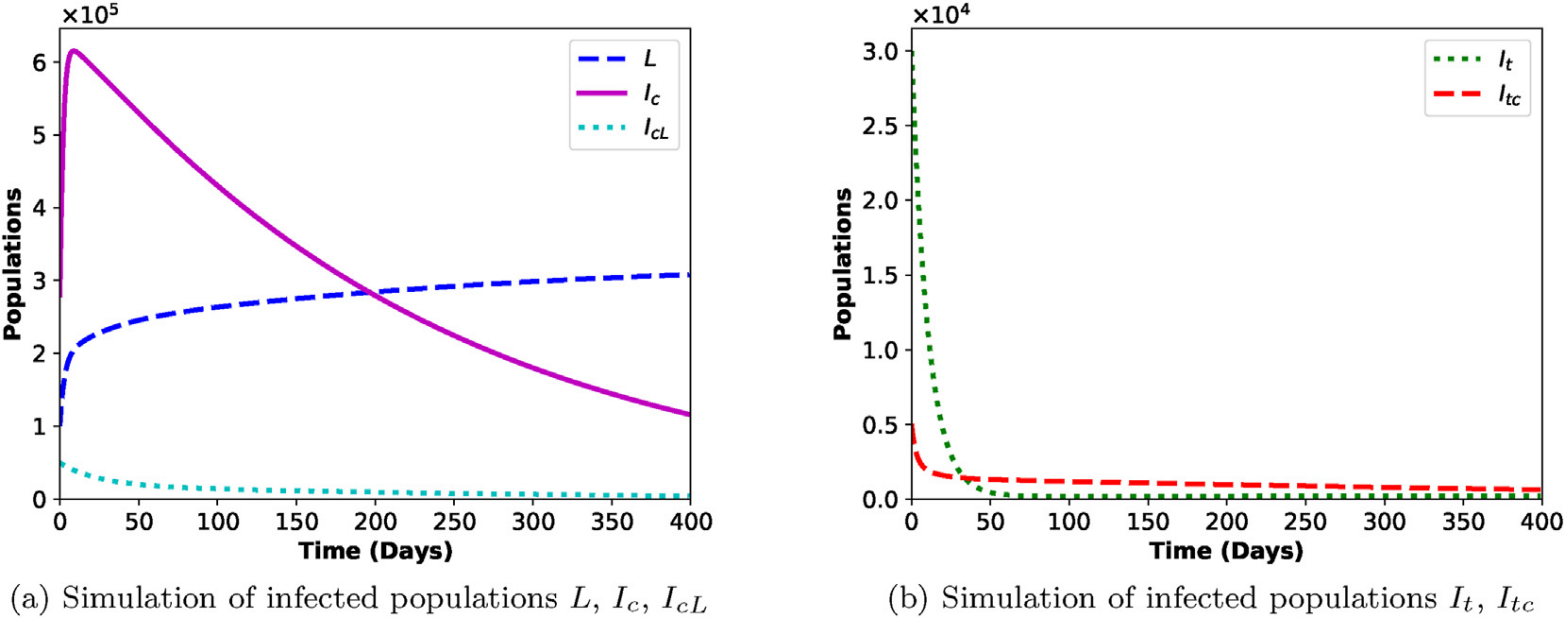
Model simulation without applying any control.

We start the numerical simulations of optimal control model (26) for the infected populations when no control strategies are applied as shown in Fig. 2. For the numerical simulations, we used the parameter values given in Table 2 which results the basic reproduction number $R_{0}=\max\left\{R_{vC},R_{0T}\right\}=\max\left\{0.82,0.93\right\}=0.93<1$. As we can observed from Fig. 2, the solutions are not converging to zero. This is because of the existence of backward bifurcation. That is, for *R*_0_ < 1, we can not eliminate the disease from the community.

**Fig. 3:**
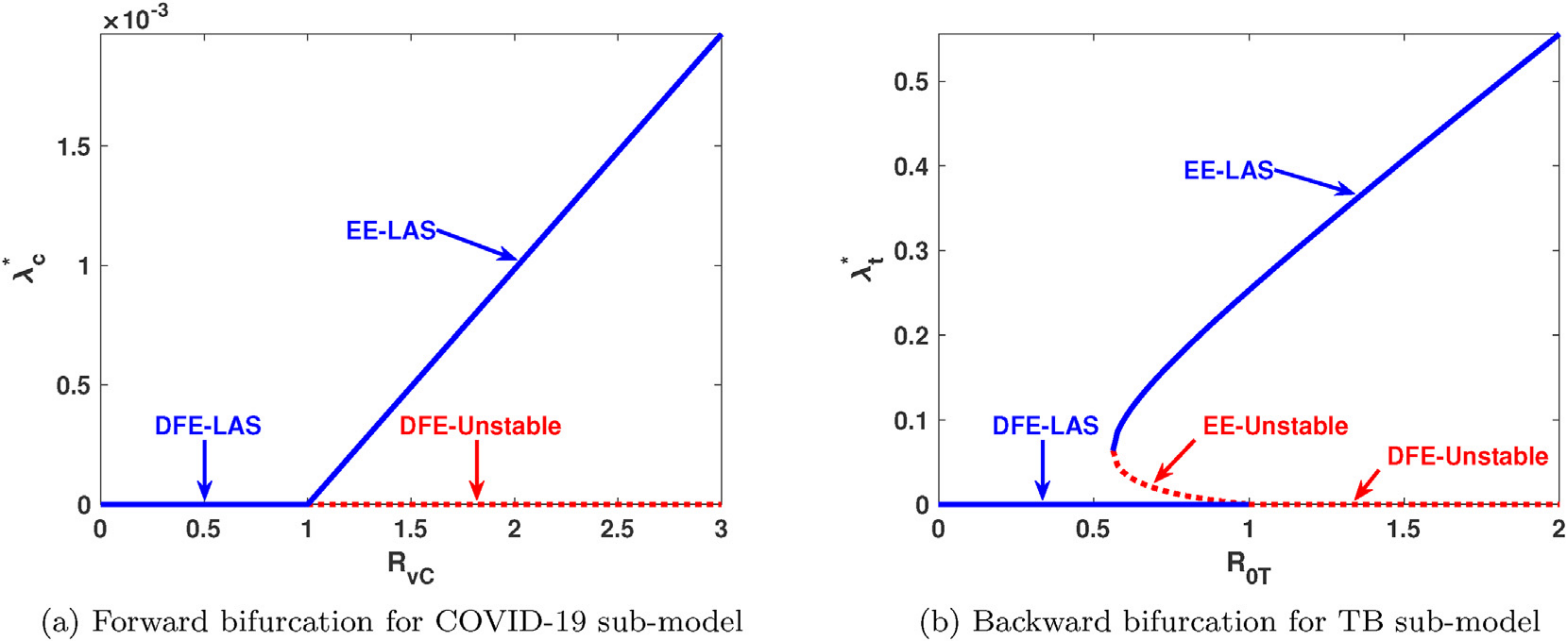
Forward/transcritical and backward/subcritical bifurcation for the force of infection $\left(\lambda_{c}^{*}\right)$ of the COVID-19 sub-model (3) and $\left(\lambda_{t}^{*}\right)$ of the TB sub-model (14), respectively.

Fig. 3 shows the forward and backward bifurcation diagrams. Fig. 3a shows that the system (3) undergoes the transcritical bifurcation at *R*_*vC*_ and changes the stability from the disease free equilibrium (DFE) to the endemic equilibrium (EE). Precisely, the DFE is stable when *R*_*vC*_ < 1 and DFE becomes an unstable equilibrium and EE becomes a stable equilibrium for the model (3) when *R*_*vC*_ > 1. Further, Fig. 3b shows that the system (14) undergoes the backward bifurcation at *R*_0*T*_ where a stable endemic equilibrium co-exists with a stable disease-free equilibrium for *R*_0*T*_ < 1. Clearly, this phenomenon has significant public health consequences, as it makes the classical requirement of the associated basic reproduction number being less than unity to be necessary, but not sufficient to eradicate the disease.

(a) Forward bifurcation for COVID-19 sub-model (b) Backward bifurcation for TB sub-model

The impact of implementing various possible control strategies on the codynamics of COVID-19 and tuberculosis disease is investigated as follows.

#### 5.1.1 Strategy A: prevention against COVID-19 (*u*_1_ ≠ 0)

**Fig. 4:**
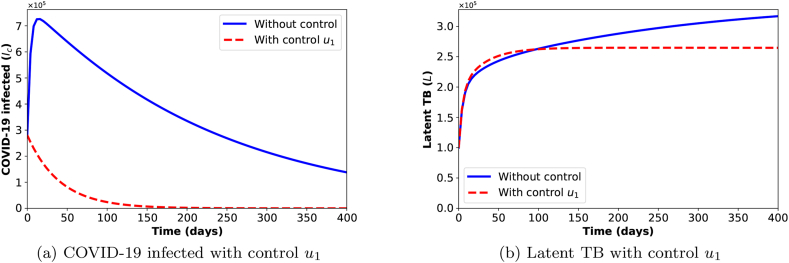
Model simulation of (a) COVID-19 infected, (b) Latent TB when the control *u*_1_ is implemented.

**Fig. 5:**
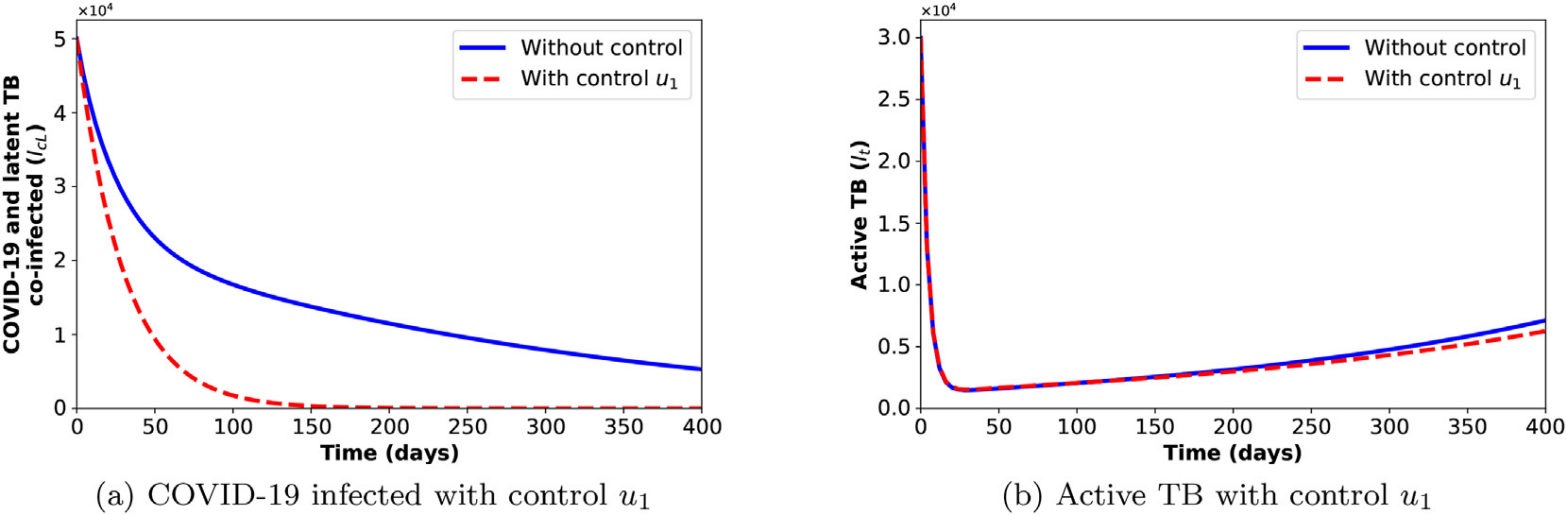
Model simulation of (a) COVID-19 and latent TB co-infected, (b) Active TB when the control *u*_1_ is implemented.

**Fig. 6:**
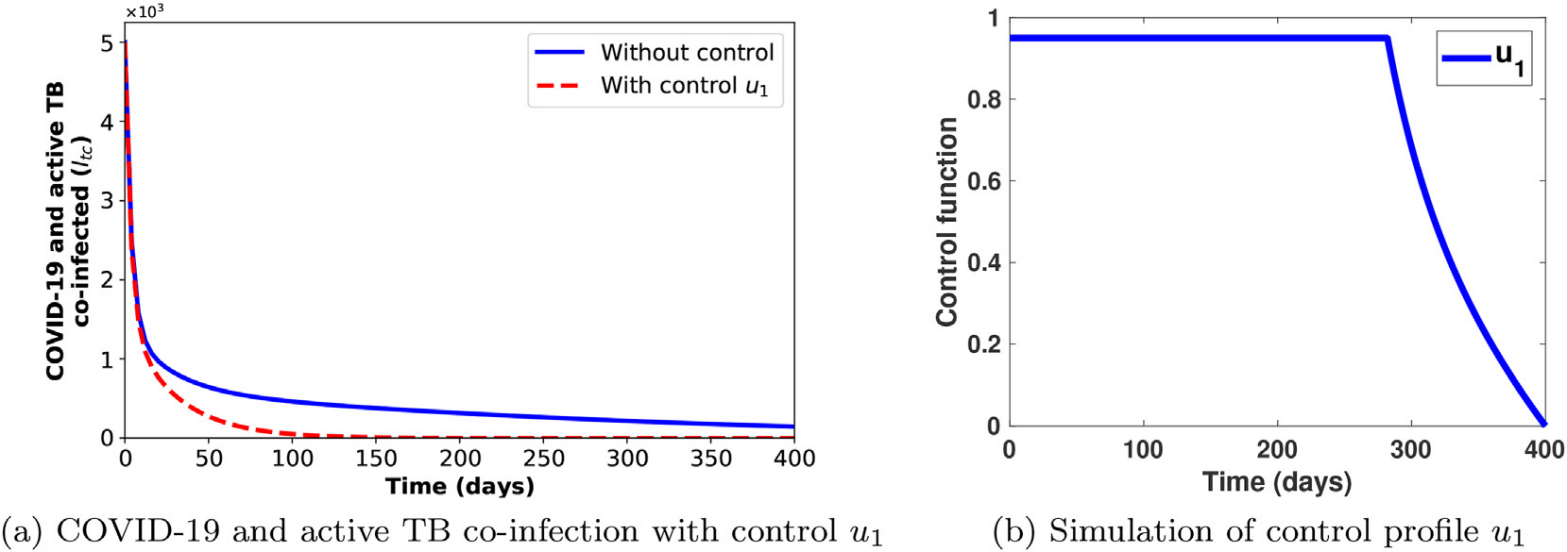
Model simulation of (a) COVID-19 and active TB co-infection when the control *u*_1_ is implemented, (b) associated control function *u*_1_.

The simulations of the optimal control system (26) when the control strategy COVID-19 prevention (*u*_1_) is implemented, are presented in Fig. 4, Fig. 5, Fig. 6. As shown in Fig. 4a, the control *u*_1_ greatly reduce the COVID-19 infected from the community within the intervention time. Further, from Fig. 5, Fig. 6a, it is demonstrated that the COVID-19 prevention strategy have a significant effect on reducing the co-infected individuals. From Fig. 4, Fig. 5b, one can also observe that the COVID-19 prevention method like face-mask usage has an effect in reducing individuals become TB infected. The control profile *u*_1_ representing the effect of prevention against COVID-19 control strategy is depicted in Fig. 6b. When prevention against COVID-19 is the selected strategy, it is observed that the prevention against COVID-19 (*u*_1_) should be implemented optimally throughout the intervention period, and then it will start decreasing after about 280 days.

#### 5.1.2 Strategy B: vaccination against COVID-19 (*u*_2_ ≠ 0)

**Fig. 7:**
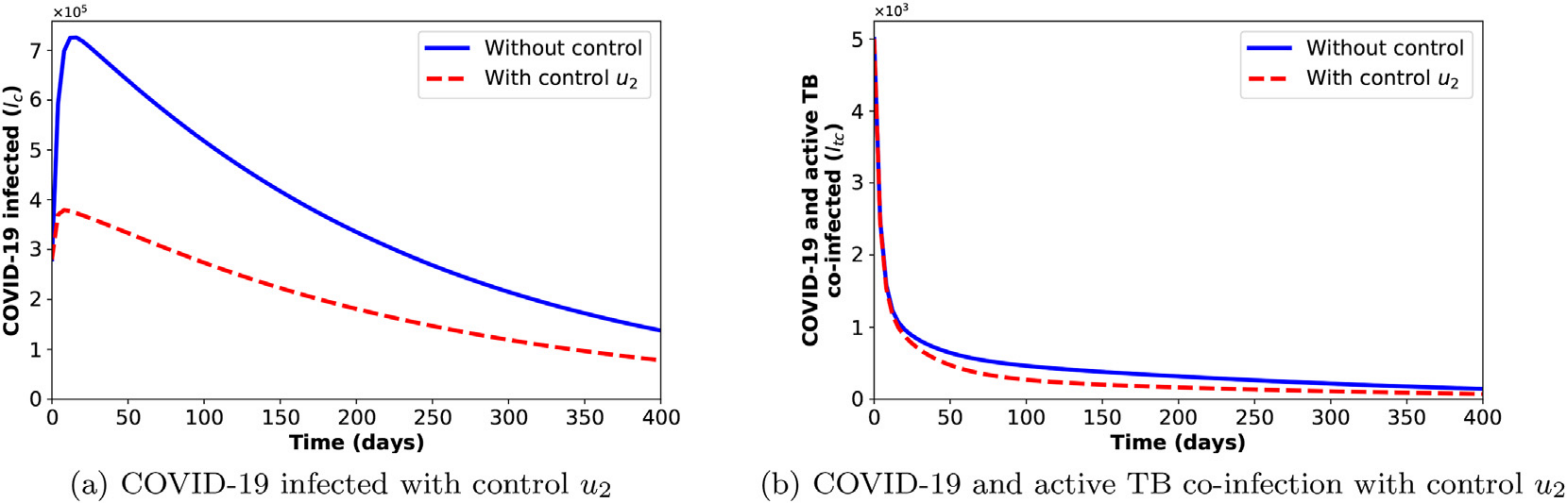
Model simulation of (a) COVID-19 infected, (b) COVID-19 and active TB co-infection when the control *u*_2_ is implemented.

**Fig. 8:**
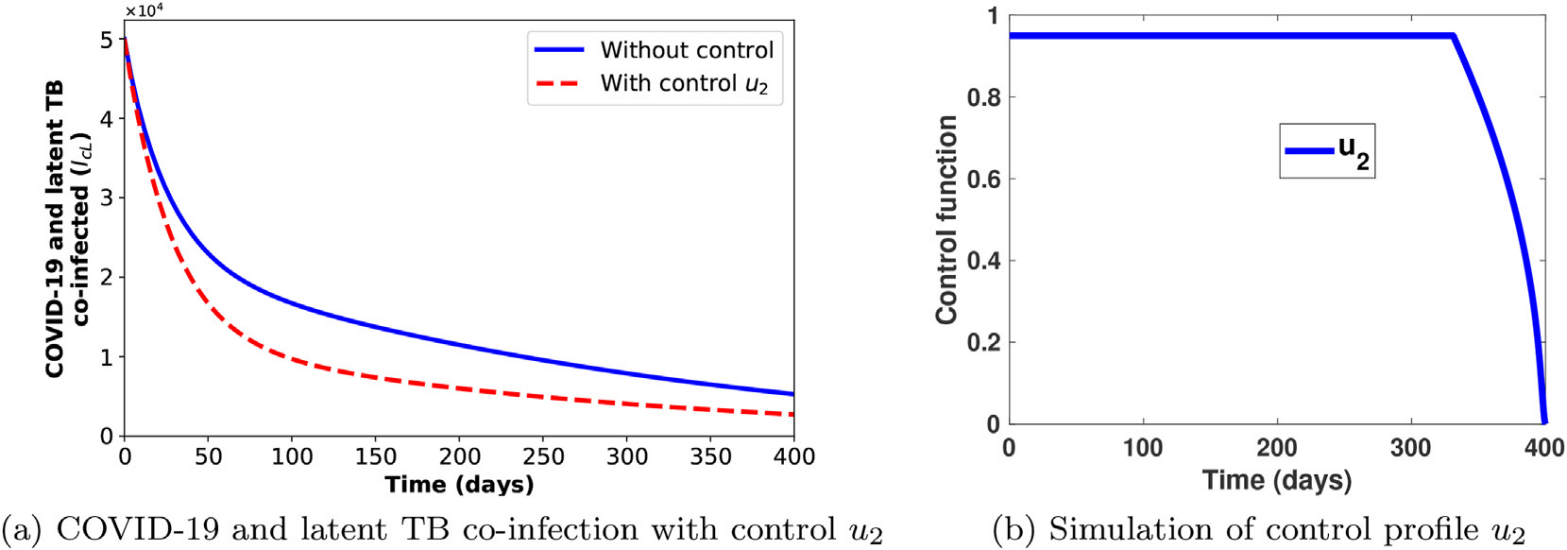
Model simulation of (a) COVID-19 and latent TB co-infection when the control *u*_2_ is implemented, (b) associated control function *u*_2_.

In this case, the COVID-19 vaccination control strategy (*u*_2_) is implemented to minimize the disease burden from the population as presented in Fig. 7, Fig. 8. From Fig. 7a, one can observe that when the control *u*_2_ is considered, the optimal strategy provide a reduction of 3 × 10^5^ COVID-19 infected individuals. The corresponding control profile *u*_2_ is also depicted in Fig. 8b. As shown in Fig. 8b, to minimize the cost functional, the optimal control effort *u*_2_ decreases from the maximum to the minimum in the final time. That is, COVID-19 vaccination *u*_2_ should be implemented optimally throughout the intervention period, and then it will start decreasing after about 320 days.

#### 5.1.3 Strategy C: TB treatment (*u*_3_ ≠ 0)

**Fig. 9:**
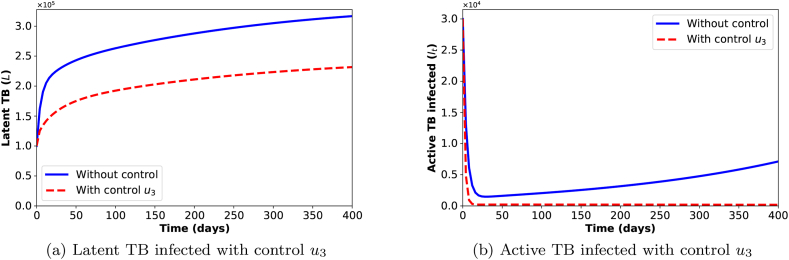
Model simulation of (a) Latent TB infected, (b) Active TB infected when the control *u*_3_ is implemented.

**Fig. 10:**
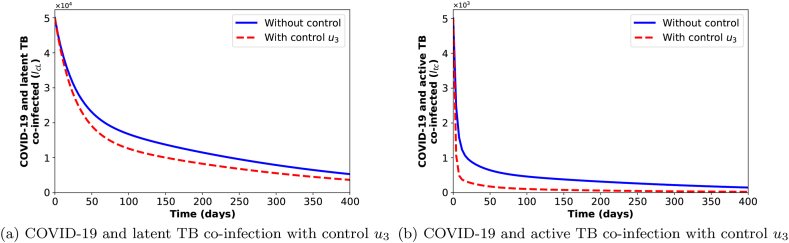
Model simulation of (a) COVID-19 and latent TB co-infection, (b) COVID-19 and active TB co-infection when the control *u*_3_ is implemented.

**Fig. 11:**
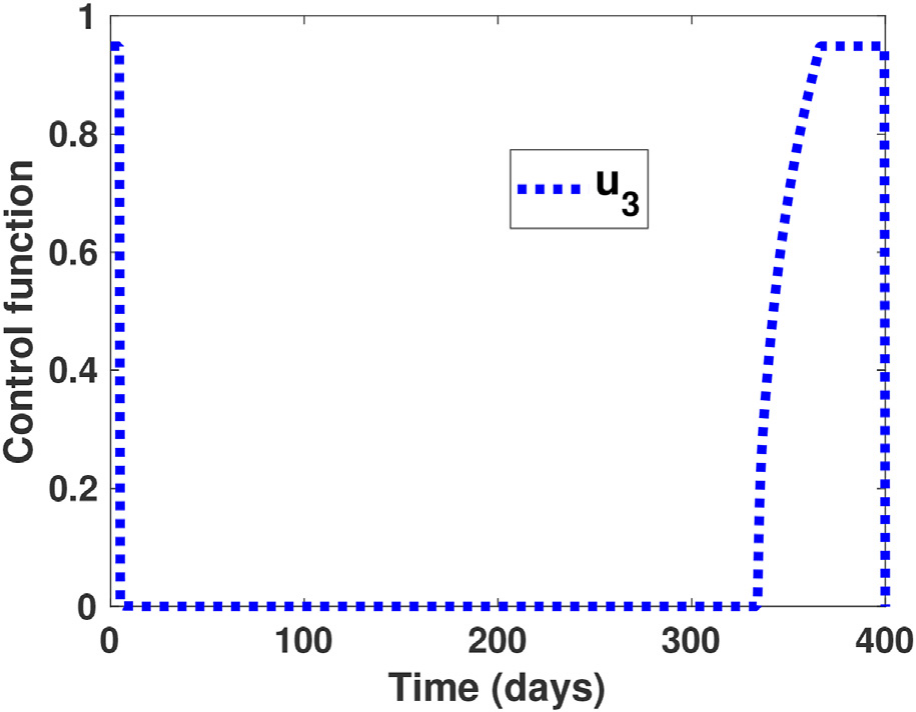
Simulation of control profile *u*_3_.

In this case, the TB treatment control strategy *u*_3_ is implemented to observe its effects in minimizing the number of TB infected and co-infected as illustrated in Fig. 9, Fig. 10. From Fig. 9a, one can observe that when the control *u*_3_ is considered, the optimal strategy provide a reduction of 1 × 10^5^ latent TB infected individuals. Similarly, from Fig. 9b, it can be seen that the TB treatment control *u*_3_ have a significant effect on reducing actively infected TB. Further, the effects of TB treatment on reducing the coinfected persons are also depicted in Fig. 10a and b. The corresponding control profile *u*_3_ is also portrayed in Fig. 11, and it can be seen that the TB treatment control *u*_3_ is optimally implemented at the onset of the epidemic, but drastically decreases and only picks up again from day 330 and become effective throughout the simulation period.

#### 5.1.4 Strategy D: COVID-19 prevention and vaccination (*u*_1_, *u*_2_ ≠ 0)

**Fig. 12:**
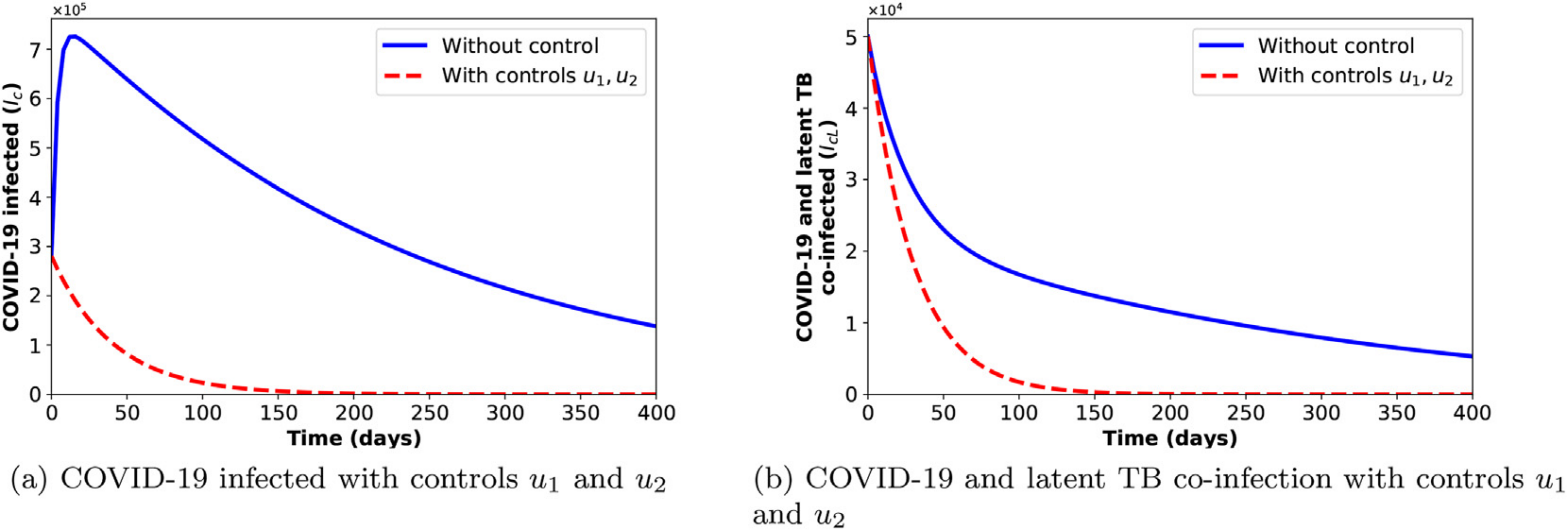
Model simulation of (a) COVID-19 infected, (b) COVID-19 and latent TB co-infection when the controls *u*_1_ and *u*_2_ are implemented.

**Fig. 13:**
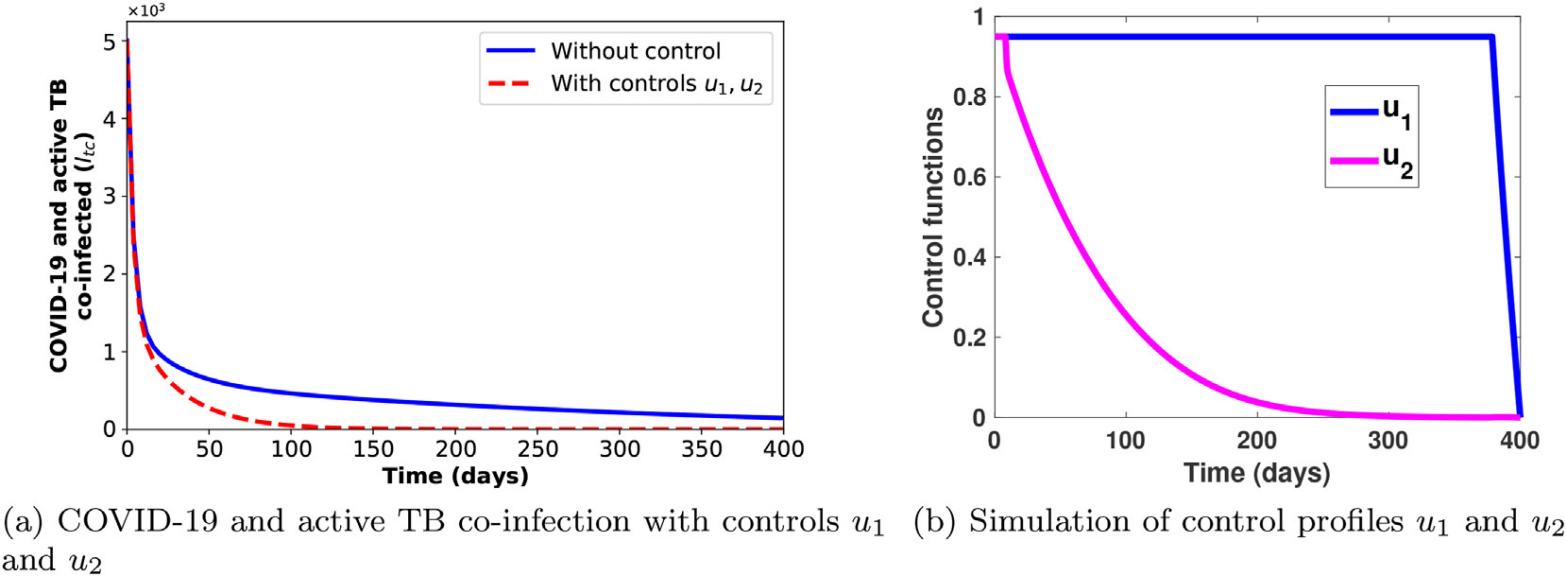
Model simulation of (a) COVID-19 and active TB co-infection when the controls *u*_1_ and *u*_2_ are implemented, (b) associated control functions *u*_1_ and *u*_2_.

In Fig. 12, Fig. 13, the implementation of control strategy that combines COVID-19 prevention and vaccination (*u*_1_ ≠ 0, *u*_2_ ≠ 0) is implemented. From Fig. 12a as expected, with implementation of this strategy a significant number of COVID-19 infected persons are reduced. Besides, it has a positive impact on reducing the co-infected individuals as shown in Fig. 12, Fig. 13a. The corresponding combined effect of controls *u*_1_ and *u*_2_ are also graphically depicted in Fig. 13b. When the combination of these control strategy are selected, one notes that COVID-19 prevention (control *u*_1_) should be implemented optimally and will start decreasing after about one year throughout the intervention period, while COVID-19 vaccination *u*_2_ uptake will start decreasing after about 20 days.

#### 5.1.5 Strategy E: COVID-19 prevention and TB treatment (*u*_1_, *u*_3_ ≠ 0)

**Fig. 14:**
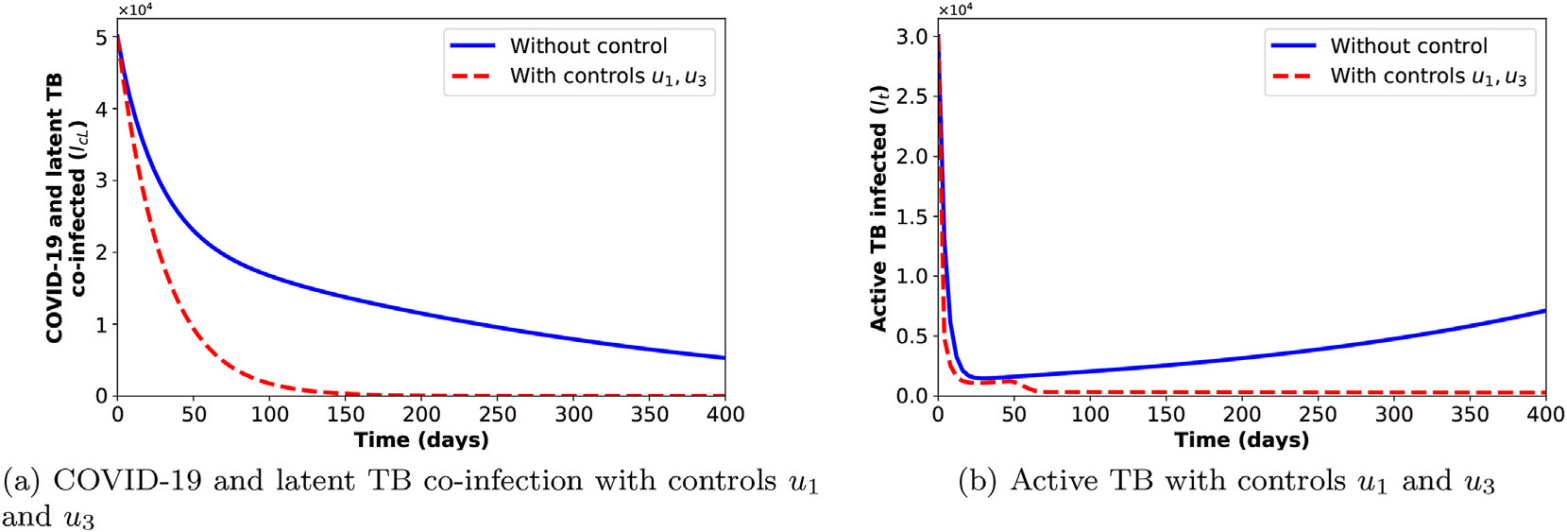
Model simulation of (a) COVID-19 and latent TB co-infection, (b) Active TB infected when the controls *u*_1_ and *u*_3_ are implemented.

**Fig. 15:**
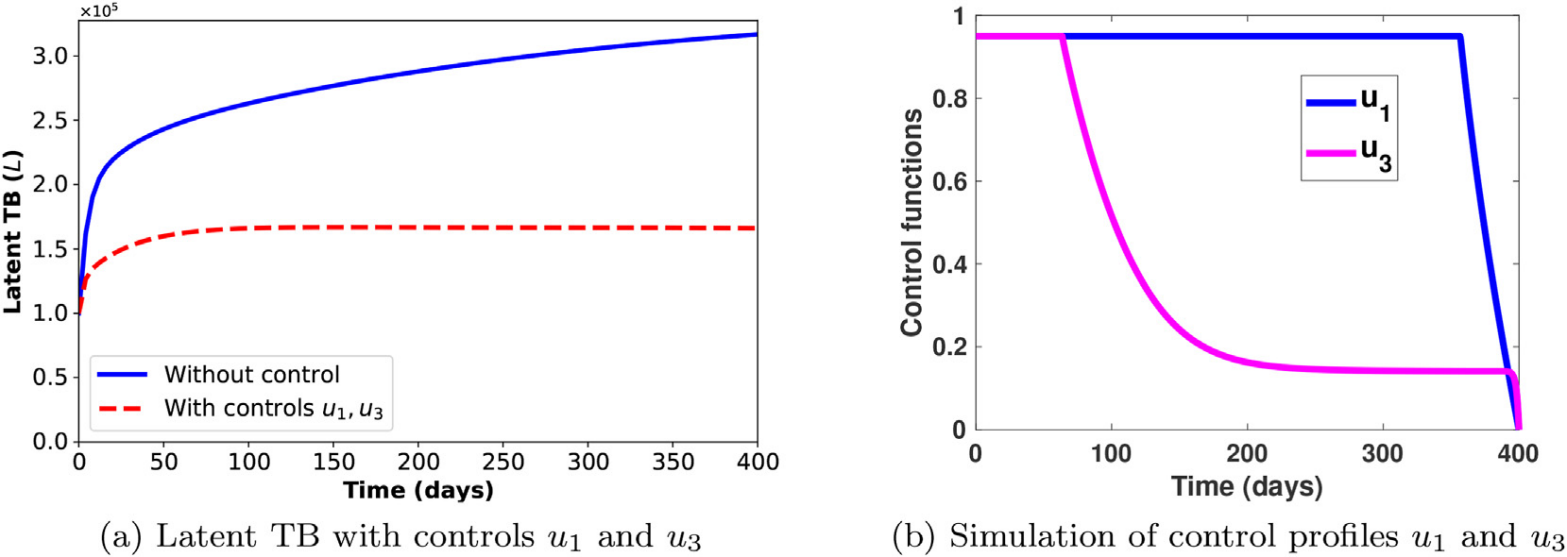
Model simulation of (a) Latent TB when the controls *u*_1_ and *u*_3_ are implemented, (b) associated control functions *u*_1_ and *u*_3_.

In Fig. 14, Fig. 15, the implementation of the control strategy that combines COVID-19 prevention and TB treatment (*u*_1_ ≠ 0, *u*_3_ ≠ 0) is implemented. From Fig. 14a, with implementation of this strategy a significant number of COVID-19 and latent TB co-infected persons are reduced. Besides, it has a positive impact on reducing active TB infected individuals as shown in Fig. 14b. Furthermore, from Fig. 15a one can observe that, when this control strategies are considered, the optimal strategy provides a reduction of 2 × 10^5^ latent TB infected cases. The corresponding combined effect of controls *u*_1_ and *u*_3_ are also graphically depicted in Fig. 15b. When the combination of these control strategy are selected, one notes that COVID-19 prevention (control *u*_1_) should be implemented optimally and will start decreasing after about 350 days throughout the intervention period, while TB treatment *u*_3_ uptake will start decreasing after about 60 days.

## 6 Conclusions

In this paper, we presented a new mathematical model for the co-dynamics of COVID-19 and TB which incorporates the COVID-19 vaccination and exogenous reinfection for TB. The model's basic properties, including the invariant region, positivity, and boundedness of solutions, are given. Furthermore, theoretical findings such as stability and bifurcation analysis of each sub-models equilibria are discussed. It is shown that the DFE is locally asymptotically stable for both COVID-19 and TB sub-models when the associated basic reproduction numbers *R*_*vC*_ < 1 and *R*_0*T*_ < 1, and unstable otherwise. The existence and stability of the endemic equilibrium point was determined for both sub models. Besides, with the help of centre manifold theory, we also proved that the COVID-19 sub-model exhibit forward bifurcation, while the TB sub-model exhibit the backward bifurcation. That is, the incorporation of exogenous reinfection into our model causes effects by allowing the occurrence of backward bifurcation for *R*_0_ < 1 and the exogenous reinfection rate larger than a threshold, (i.e., *η* > *η*∗). Based on the occurrence of backward bifurcation for TB sub-model, and since the dynamics of COVID-19 and TB co-infection model is driven by that of its sub-model, then the model (1) exhibit the phenomenon of backward bifurcation. Thus, the possible implication of this phenomenon is that reducing *R*_0_ < 1 may not be sufficient to eliminate the disease burden from the community. Further, we observed that when *η* = 0, system (1) reduces to the coinfection model with out exogenous reinfection. In this case, the backward bifurcation phenomenon will not occur and the disease elimination from the community is possible for *R*_0_ < 1.

The proposed co-infection model is then extended to included three control measures. The conditions for the existence of optimal control and the optimality system for the control induced model are established using Pontryagin's maximum principle. Furthermore, different numerical simulations of the control induced model are also performed to observe the effects of the control strategies by using model parameters given in Table 2. To further understand the dynamical interactions between COVID-19 and TB with and without optimal control, simulations are carried out in two scenarios, one with single control and other the combination of two controls. Results of these two scenarios are compared with the model without control and it reveals that the infected sub-population reduces significantly when combinations of the controls are applied. When COVID-19 vaccination is considered as control strategy, the optimal strategy provide a reduction of significant number of COVID-19 infected cases. Further, the combination of COVID-19 prevention and vaccination control strategy greatly mitigates new co-infection cases.

## Data availability

All datasets generated in this study can be recovered from information available within the article.

## Declaration of competing interest

The authors declare that there is no conflict of interest.

## Appendix A Computation of basic reproduction number

The basic reproduction number for system (14) can be calculated using the approach in (Van den Driessche & Watmough, 2002). Considering the infected compartments $x={\left(x_{1},x_{2}\right)}^{T}={\left(L,I_{t}\right)}^{T}$, system (14) can be written as

$$\dot{x}=f\left(x\right)=F\left(x\right)-V\left(x\right),$$

where

$$F=\left[\begin{array}{c} \beta_{t}\frac{\left(S+\varphi R_{t}\right)I_{t}}{N}\\ 0 \end{array}\right]\;\text{and}\;V=\left[\begin{array}{c} \eta \lambda_{t}L+\left(\varphi_{t}+\mu \right)L\\ -\varphi_{t}L-\eta \lambda_{t}L+\left(r_{t}+\mu +\delta_{2}\right)I_{t} \end{array}\right].$$

Consequently, the current infection terms *F* and the remaining transfer terms *V* are given by

$$F=\frac{\partial F}{\partial x_{i}}{|}_{E_{t}^{0}}=\left[\begin{array}{cc} 0 & \beta_{t}\\ 0 & 0 \end{array}\right]\;\text{and}\;V=\frac{\partial V}{\partial x_{i}}{|}_{E_{t}^{0}}=\left[\begin{array}{cc} \varphi_{t}+\mu & 0\\ -\varphi_{t} & r_{t}+\mu +\delta_{2} \end{array}\right],$$

where *i* = 1, 2. The inverse of the matrix *V* is given by

$$V^{-1}=\left[\begin{array}{cc} \frac{1}{\varphi_{t}+\mu } & 0\\ \frac{\varphi_{t}}{\left(\varphi_{t}+\mu \right)\left(r_{t}+\mu +\delta_{2}\right)} & \frac{1}{r_{t}+\mu +\delta_{2}} \end{array}\right].$$

Thus, the next generation matrix *FV*^−1^ is given by

$$FV^{-1}=\left[\begin{array}{cc} \frac{\beta_{t}\varphi_{t}}{\left(\varphi_{t}+\mu \right)\left(r_{t}+\mu +\delta_{2}\right)} & \frac{\beta_{t}}{r_{t}+\mu +\delta_{2}}\\ 0 & 0 \end{array}\right].$$

Taking the dominant eigenvalue of *FV*^−1^, the basic reproductive number for TB sub-model (14) is given by

$$R_{0T}=\left(\frac{\beta_{t}}{r_{t}+\mu +\delta_{2}}\right)\left(\frac{\varphi_{t}}{\varphi_{t}+\mu }\right).$$
